# BCAS-3 is required for the progression of autophagosome formation to degrade paternal mitochondria in *Caenorhabditis elegans*

**DOI:** 10.1016/j.isci.2026.116345

**Published:** 2026-06-11

**Authors:** Takuya Norizuki, Taeko Sasaki, Yuji Suehiro, Shohei Mitani, Waka Kojima, Koji Yamano, Noriyuki Matsuda, Miyuki Sato

**Affiliations:** 1 Laboratory of Molecular Membrane Biology, Institute for Molecular and Cellular Regulation, Gunma University, Maebashi, Gunma, Japan; 2 Department of Physiology, Tokyo Women’s Medical University School of Medicine, Shinjuku-ku, Tokyo, Japan; 3 Department of Biomolecular Pathogenesis, Medical Research Laboratory, Institute of Integrated Research, Institute of Science Tokyo, Bunkyo-ku, Tokyo, Japan; 4 Intracellular Quality Control Project, Tokyo Metropolitan Institute of Medical Science, Setagaya-ku, Tokyo, Japan

**Keywords:** cell biology, developmental biology, molecular biology

## Abstract

In the nematode *Caenorhabditis elegans*, autophagy degrades paternal mitochondria after fertilization to ensure the maternal inheritance of mitochondrial DNA. We previously showed that the autophagy adaptor ALLO-1 is first targeted to paternal mitochondria and then recruits the autophagy machinery. However, the mechanisms underlying local autophagosome formation remain unclear. Here, our forward genetic screen identified a WD40 repeat domain-containing protein, BCAS-3, and its interactor, PHAF-1, as essential factors for paternal mitochondrial degradation. After fertilization, BCAS-3 and PHAF-1 are recruited to the paternal mitochondria, and the loss of these genes impairs the progression of autophagosome formation. We further show that BCAS-3 recruitment is regulated downstream of the WD40 repeat domain-containing core autophagy proteins, ATG-18 and EPG-6, but BCAS-3 also contributes to further ATG-18 accumulation around paternal mitochondria. These findings suggest that the interplay between BCAS-3 and ATG-18 underlies the progression of autophagosome formation during paternal mitochondrial degradation.

## Highlights

• Our genetic screen identifies BCAS-3 and PHAF-1 in paternal mitochondrial degradation
• BCAS-3 and PHAF-1 are recruited to paternal mitochondria after fertilization
• ATG-18 and EPG-6 are required for BCAS-3 recruitment to paternal mitochondria
• BCAS-3 promotes ATG-18 accumulation around paternal mitochondria

## Graphical abstract

**Figure:**
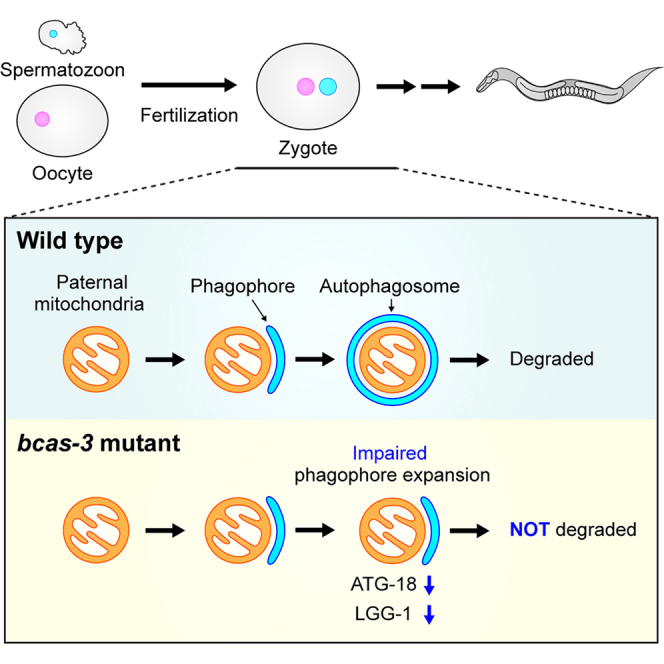

## Introduction

Mitochondria are semi-autonomous organelles that possess their own DNA, which is pivotal for mitochondrial function and is inherited maternally in most organisms. We and others have shown that macroautophagy, hereafter referred to simply as autophagy, eliminates paternal mitochondria after fertilization, thereby ensuring maternal inheritance of mitochondrial DNA in the nematode *Caenorhabditis elegans*.^1^^,^^2^^,^^3^ Similar degradation has also been observed in other species, including mice.^4^^,^^5^^,^^6^ In *C*. *elegans*, other sperm-derived organelles, membranous organelles (MOs), which are the post-Golgi compartments required for fertilization, are also degraded by autophagy.^1^^,^^2^ These autophagic degradations of sperm-derived organelles are termed allophagy (allogeneic [non-self] organelle autophagy). In *C*. *elegans*, the autophagy adaptor ALLO-1 (allophagy-1) is targeted to these paternal organelles and then recruits ATG (autophagy-related) proteins via direct interactions with the ATG8 family protein LGG-1 and the Atg11/FIP200 homolog EPG-7 to form a double membrane structure called an autophagosome, which sequesters paternal organelles.^7^^,^^8^ However, the detailed molecular mechanisms underlying this local autophagosome formation remain unclear.

In autophagy, a flat membrane cisterna, termed the phagophore (or isolation membrane), expands to sequester cytoplasmic components and eventually forms an autophagosome. Conversion of phosphatidylinositol to phosphatidylinositol 3-phosphate (PI3P) by the phosphatidylinositol 3-kinase VPS34 complex is pivotal for autophagosome formation and recruits PI3P-binding proteins, such as the β-propeller that binds polyphosphoinositides (PROPPIN) family proteins, to the autophagosome formation site.^9^ Mammals possess four PROPPIN proteins, WIPI1–4 (WD repeat protein interacting with phosphoinositides 1–4), which bind to PI3P through the WD40 repeat domain. WIPI2 regulates autophagosome formation through ATG16L1 recruitment, which promotes the conjugation of ATG8 family proteins with phosphatidylethanolamine on the phagophore, whereas WIPI3/4 regulates autophagosome size and autophagosome-lysosome fusion.^10^^,^^11^^,^^12^^,^^13^ *C*. *elegans* possesses two PROPPINs, ATG-18 (WIPI1/2 group) and EPG-6 (WIPI3/4 group) (Figures S1A and S1B), which have been reported to function at distinct steps of autophagosome formation during the degradation of the germ granule (P granule in *C*. *elegans*) component PGL-1 in somatic cells.^14^ Recently, we and others identified that another PI3P-binding WD40 repeat protein BCAS3 (BCAS3 microtubule-associated cell migration factor)/Rudhira is involved in autophagy in human cells and the slime mold *Dictyostelium discoideum*.^15^^,^^16^ Furthermore, such as PROPPIN family proteins, loss of BCAS3 function is linked to human neurodevelopmental disorders,^17^^,^^18^^,^^19^^,^^20^ although the direct link between autophagic activity and disease pathogenesis remains to be elucidated. In addition to BCAS3, its interactor PHAF1/KinkyA/MYTHO, which harbors an uncharacterized UPF0183 domain but no other domains with known functions, is also involved in autophagy in human cells, *D*. *discoideum,* and muscle cells of mammals and *C*. *elegans*.^15^^,^^16^^,^^21^^,^^22^ However, it remains unclear at which steps of autophagosome formation BCAS3 and PHAF1 are involved, such as membrane nucleation or expansion. Furthermore, although previous studies suggested that they function together with WIPI proteins,^15^^,^^22^ their functional relationship with these proteins remains unclear.

In this study, we performed forward genetic screening and identified BCAS-3 and PHAF-1 as essential factors for the autophagic degradation of paternal mitochondria. BCAS-3 and PHAF-1 are recruited to paternal mitochondria, and the loss of these functions leads to defects in the progression of autophagosome formation. Genetic analyses have also revealed that BCAS-3 recruitment is regulated downstream of PROPPIN proteins ATG-18 and EPG-6, but BCAS-3 is also important for proper ATG-18 accumulation, suggesting that the interplay between ATG-18 and BCAS-3 promotes the progression of local autophagosome formation.

## Results

### BCAS-3 and PHAF-1 are required for paternal mitochondrial degradation

**Figure 1:**
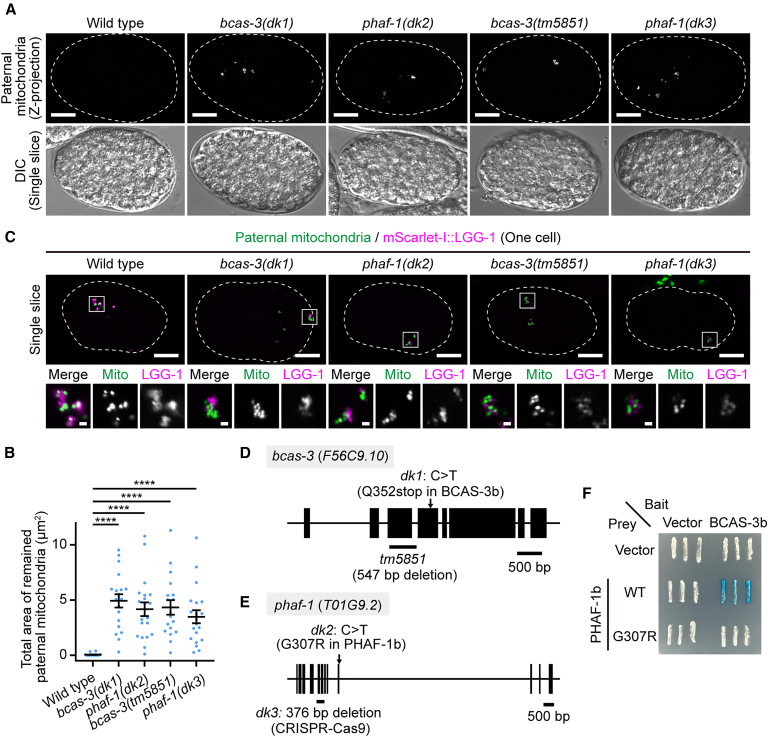
BCAS-3 and PHAF-1 are required for paternal mitochondrial degradation (A and B) Defects in the degradation of paternal mitochondria in *bcas-3* and *phaf-1* mutants. Embryos at approximately the 64–100 cell stage were observed, and the total area of persistent paternal mitochondria in these embryos was quantified. Error bars represent the mean ± SEM. *n* = 18 (wild type and *bcas-3(tm5851)*), 19 (*bcas-3(dk1)*) and 20 (*phaf-1(dk2)* and *phaf-1(dk3)*) embryos. Statistical differences were determined using the Mann–Whitney U test followed by Holm correction. ∗∗∗∗*p* < 0.0001. (C) Defects in LGG-1 recruitment around paternal mitochondria in *bcas-3* and *phaf-1* mutants. 18–21 zygotes from pronucleus migration to the completion of the first cytokinesis were observed, and representative images are shown. Z-projection images of the boxed region are shown in Figure S2A. (D and E) Mutation sites of *dk1* and *dk2* in the *bcas-3* and *phaf-1* genes. The deletion sites in *tm5851* and *dk3* are also shown. *tm5851* was provided by the National Bioresource Project, and *dk3* was generated using the CRISPR-Cas9 system. (F) Interaction between BCAS-3 and PHAF-1 in a yeast two-hybrid analysis. Paternal mitochondria were visualized using HSP-6::GFP driven by the *spe-11* promoter. Dashed lines represent the outlines of eggshells (A) or zygotes (C). Scale bars, 10 μm (A and upper panels in C) and 1 μm (lower panels in C). See also Figures S1 and S2.

To dissect the mechanisms underlying allophagy, we performed genetic screening to isolate mutants defective in paternal mitochondrial degradation. The *C*. *elegans* strain GK2140, which expresses HSP-6::GFP and mScarlet-I::LGG-1 to label sperm mitochondria^1^ and autophagosome-related structures such as phagophores in the germline,^1^ respectively, was mutagenized using ethyl methanesulfonate (EMS). Subsequently, we observed paternal mitochondria in embryos using a confocal microscope and isolated mutants in which paternal mitochondria remained to be degraded during embryogenesis. To determine which step in autophagy was affected, we observed the localization of mScarlet-I::LGG-1 in the zygotes of these mutants. Among more than 6,000 EMS lines screened, we re-isolated allophagy-defective mutants previously reported, such as *allo-1* mutants.^7^ In addition, we isolated two mutants, *dk1* and *dk2*, in which paternal mitochondria remained in late-stage embryos (Figures 1A and 1B). In these mutants, mScarlet-I::LGG-1 was partially accumulated around paternal mitochondria but failed to fully sequester paternal mitochondria, as evidenced by Z-projected images (Figures 1C and S2A). This defect was different from that in mutants of genes required for allophagy initiation, such as the autophagy adaptor *allo-1* and *ATG11/FIP200* homolog *epg-7*, in which LGG-1 is not recruited to paternal mitochondria.^7^^,^^8^ These results suggest that *dk1* and *dk2* are defective in the progression rather than the initiation of autophagosome formation.

Whole-genome sequencing and complementation analyses (see STAR Methods) revealed that the mutations in *F56C9*.*10* and *phaf-1* are responsible for the allophagy defect in *dk1* and *dk2*, respectively. *F56C9*.*10* encodes the *C*. *elegans* homolog of BCAS3, which we named *bcas-3*. *C*. *elegans* BCAS-3 contains the WD40 repeat domain in the central region. In the *bcas-3(dk1)* mutant, the codon for Q352 in the WD40 repeat domain was mutated to a stop codon, resulting in the truncation of the β-propeller structure (Figures 1D and S1A). In the *phaf-1(dk2)* mutant, the highly conserved G307 in the UPF0183 domain was replaced by arginine (Figures 1E, S2B, and S2C). We also examined different mutant alleles of *bcas-3* and *phaf-1*: *bcas-3(tm5851)* and *phaf-1(dk3)*, which harbor 547 bp and 376 bp deletions, respectively (Figures 1D and 1E). Both mutants exhibited allophagy defects similar to those of *dk1* and *dk2* (Figures 1A–1C and S2A), confirming that BCAS-3 and PHAF-1 are required for the degradation of paternal mitochondria. In humans and *D*. *discoideum*, BCAS3 and PHAF1 interact and are recruited to the autophagosome formation site.^15^^,^^16^ We found that *C*. *elegans* BCAS-3 and PHAF-1 also interacted in a yeast two-hybrid analysis (Figure 1F). AlphaFold 3 predicted that PHAF-1 G307, the residue mutated in *the phaf-1(dk2)* allele, is located near the BCAS-3-binding interface (Figure S2D). Furthermore, the PHAF-1 G307R mutant failed to interact with BCAS-3 in the yeast two-hybrid analysis (Figure 1F). These results suggest that PHAF-1 G307 is important for BCAS-3 binding. Although these proteins have been reported to be involved in autophagy in several species, their precise roles in autophagy remain unclear. In *C*. *elegans*, the *phaf-1* mutant has been reported to cause a partial reduction in starvation-induced autophagy in muscle cells,^22^ whereas *F56C9*.*10/bcas-3* remained uncharacterized. Hereafter, we further analyzed the roles of BCAS-3 and PHAF-1 during allophagy. We used the *bcas-3(tm5851)* and *phaf-1(dk3)* deletion mutants for further analysis.

### BCAS-3 and PHAF-1 are required for the degradation of membranous organelles, but are less important for PGL-1 degradation

**Figure 2:**
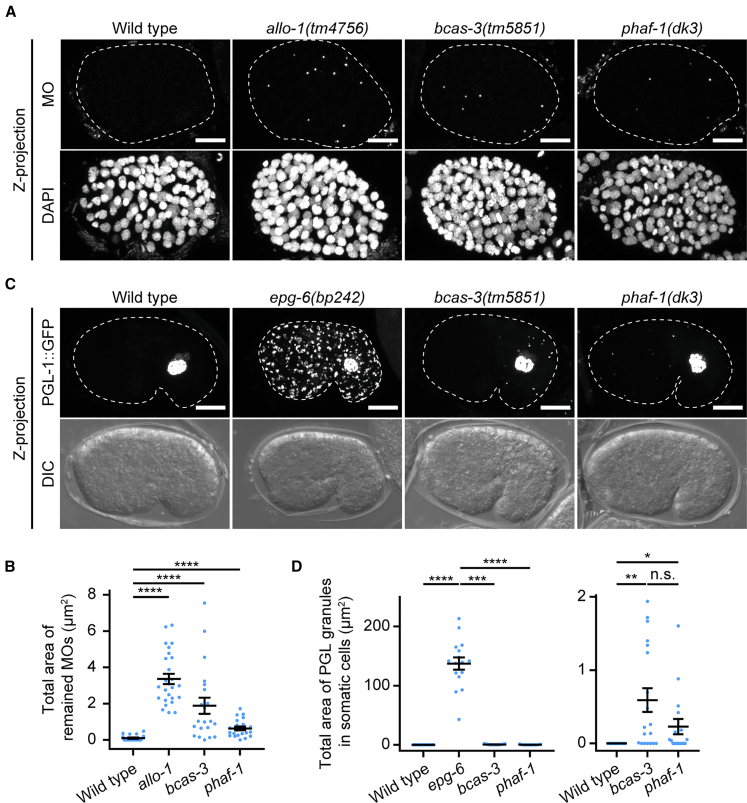
BCAS-3 and PHAF-1 are required for MO degradation but are less important for PGL-1 degradation (A and B) Persistence of MOs during embryogenesis in the *bcas-3* and *phaf-1* mutants. MOs were visualized by the antibody 1CB4, and the total area of persistent MOs was quantified. *n* = 20 (wild type), 26 (*allo-1(tm4756)*), 21 (*bcas-3(tm5851)*) and 22 (*phaf-1(dk3)*) embryos at around 100–200 cell stages. (C and D) PGL-1 distribution in the *bcas-3* and *phaf-1* mutant. Embryos from the comma to 2-fold stages were observed, and the total area of PGL-1::GFP-positive granules in somatic cells was quantified. In D, the right graph shows the left graph without data from *epg-6(bp242)*. *n* = 20 (wild type), 16 (*epg-6(bp242)*), 19 (*bcas-3(tm5851)*), and 17 (*phaf-1(dk3)*) embryos. Error bars represent the mean ± SEM. Statistical differences were determined using the Mann–Whitney U test followed by Holm correction (B) or the Kruskal–Wallis test followed by Dunn’s test with Holm correction (D). n.s.: *p* > 0.05, ∗*p* < 0.05, ∗∗*p* < 0.01, ∗∗∗*p* < 0.001, and ∗∗∗∗*p* < 0.0001. Dashed lines represent outlines of embryos. Scale bars, 10 μm.

Our genetic screening revealed that BCAS-3 and PHAF-1 are required for the degradation of paternal mitochondria (Figure 1). In addition to sperm mitochondria, MOs are degraded by allophagy after fertilization in an *allo-1*-dependent manner.^7^^,^^8^^,^^23^ To investigate whether BCAS-3 and PHAF-1 are required for MO degradation, we observed the fate of MOs using the 1CB4 antibody^24^ in these mutants. This monoclonal antibody has been previously shown to label MOs in immunoelectron microscopy and is generally used as a marker for MOs. In wild-type embryos, MOs were degraded by autophagy, and their signals were lost during embryogenesis. In contrast, similar to the *allo-1* mutant, MOs remained in the *bcas-3* and *phaf-1* mutant embryos during embryogenesis, suggesting that BCAS-3 and PHAF-1 are also required for MO degradation (Figures 2A and 2B).

We further investigated whether BCAS-3 and PHAF-1 are generally required for autophagic degradation. During embryogenesis, a germline P granule component, PGL-1, is degraded by autophagy in the somatic lineages of embryos, resulting in its exclusive distribution in germline precursor cells (Figure 2C).^25^^,^^26^ To investigate whether BCAS-3 and PHAF-1 are also required for this process, we observed the distribution of PGL-1::GFP in these mutants. In wild-type embryos, PGL-1::GFP was exclusively distributed in two primordial germ cells. In contrast, in *epg-6* mutants, PGL-1::GFP-positive granules were observed in somatic cell lineages as well, consistent with a previous report.^14^ In the *bcas-3* and *phaf-1* mutants, PGL-1 is largely degraded in their somatic lineages, although a few PGL-1::GFP-positive granules remained (Figures 2C and 2D). Taken together, BCAS-3 and PHAF-1 are required for allophagy but are less important for PGL-1 degradation in *C*. *elegans* embryos.

### BCAS-3 and PHAF-1 are recruited to paternal mitochondria after fertilization

**Figure 3:**
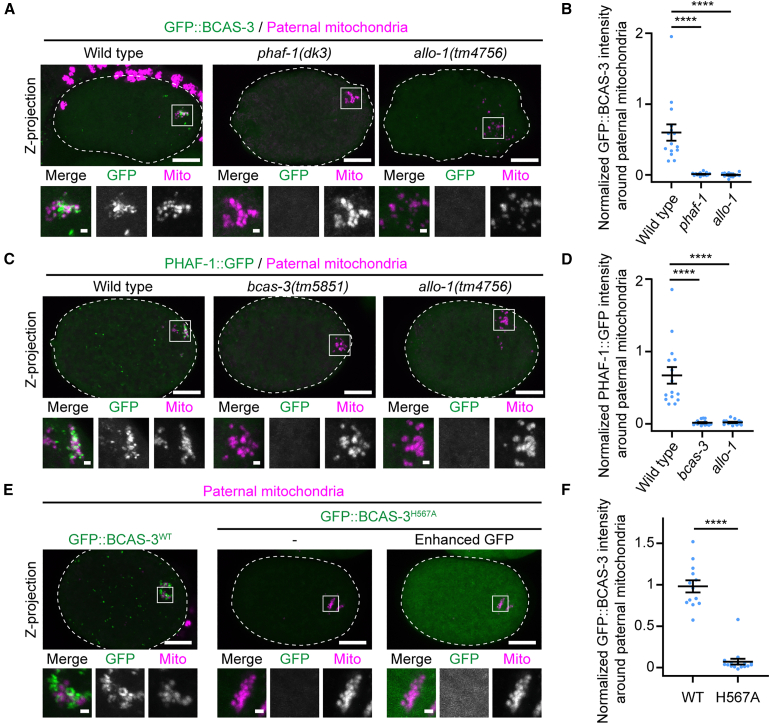
Localization of BCAS-3 and PHAF-1 during allophagy (A–D) Interdependent BCAS-3 (A) and PHAF-1 (C) localizations around paternal mitochondria. *n* = 15 zygotes per strain, from late meiosis I to pronuclear expansion stages. (E and F) PI3P-binding-dependent recruitment of BCAS-3 to paternal mitochondria. *n* = 13 (WT) and 16 (H567A) zygotes from late meiosis I to pronuclear expansion stages. The GFP::BCAS-3 (B and F) and PHAF-1::GFP (D) intensities around paternal mitochondria were normalized to the HSP-6::mCherry (paternal mitochondria) intensity in each paternal mitochondrion, and the mean value in each zygote is shown. Error bars represent the mean ± SEM. Statistical differences were determined using the Mann–Whitney U test followed by Holm correction (B and D) and the Mann–Whitney U test (F). ∗∗∗∗*p* < 0.0001. Dashed lines represent the outlines of the zygotes. Scale bars, 10 μm (upper panels) and 1 μm (lower panels). See also Figure S3.

To dissect how BCAS-3 and PHAF-1 regulate allophagy, we observed the subcellular localization of GFP-tagged BCAS-3 and PHAF-1. Expression of GFP::BCAS-3 or PHAF-1::GFP in the germline rescued the allophagy defect in each mutant, indicating that these GFP fusion proteins were functional (Figure S3A). In zygotes, both GFP::BCAS-3 and PHAF-1::GFP were localized around paternal mitochondria (Figures 3A and 3C). Consistent with the results in human cells,^15^ the localization of BCAS-3 and PHAF-1 was mutually dependent on each other. In the absence of PHAF-1, BCAS-3 failed to be recruited around paternal mitochondria, and vice versa (Figures 3A–3D). This localization also depended on ALLO-1 expression (Figures 3A–3D). Immunoblot analysis confirmed that the loss of localization was not due to reduced protein levels (Figure S3B). We also generated a strain in which a DNA fragment encoding mCherry was inserted into the *bcas-3* locus using the CRISPR/Cas9 system. Endogenously tagged BCAS-3 was expressed in the germline and showed ALLO-1-dependent localization around paternal mitochondria, consistent with the patterns observed for GFP::BCAS-3 expressed under the germline-specific promoter (Figures S3C and S3D).

Human and *D*. *discoideum* BCAS3 interact with PI3P *in vitro*, and this interaction is required for its localization in human cells.^15^^,^^16^ To investigate whether PI3P binding is required for its recruitment to paternal mitochondria, we replaced H567 with alanine in BCAS-3, because the corresponding residue of *Kluyveromyces lactis* PROPPIN Hsv2 is critical for its PI3P binding (Figure S3E).^27^ In contrast to wild-type BCAS-3 (BCAS-3^WT^), GFP::BCAS-3^H567A^ was not recruited to paternal mitochondria and was dispersed in the cytoplasm (Figures 3E and 3F). We verified that GFP::BCAS-3^H567A^ was expressed in adult hermaphrodites by immunoblotting, although its expression level was lower than that of GFP::BCAS-3^WT^ (Figures S3F). To exclude the possibility that the weak recruitment of the mutant protein to paternal mitochondria was missed, its localization was examined with increased brightness; however, little recruitment of the mutant protein to paternal mitochondria was observed (Figure 3E). These results suggest that PI3P binding to BCAS-3 is required for its localization during allophagy.

**Figure 4:**
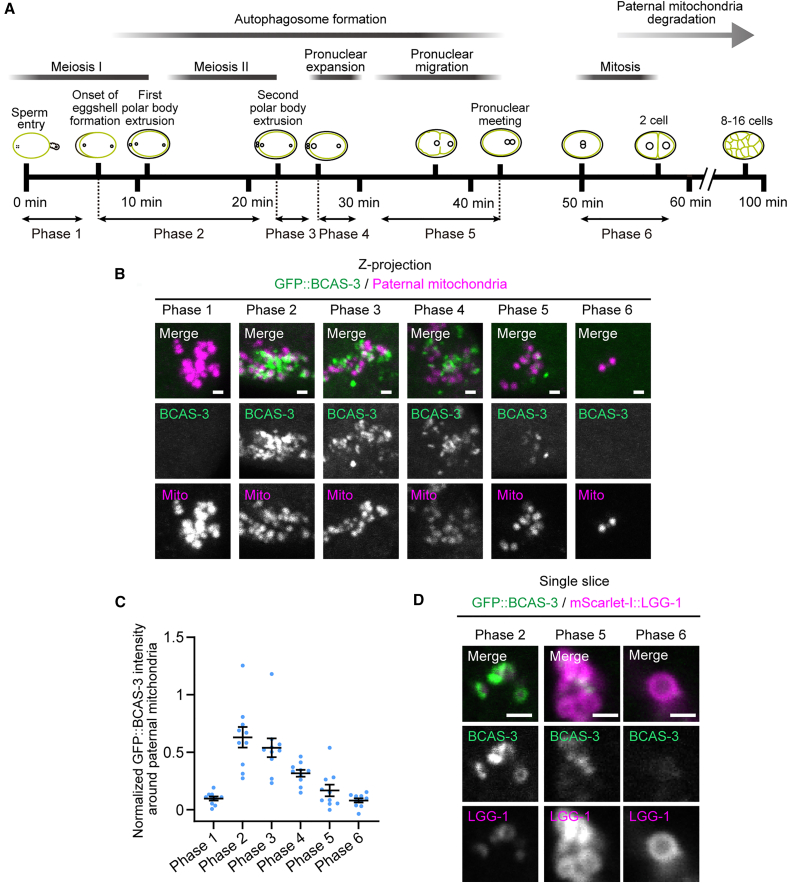
Temporal changes in GFP::BCAS-3 signal around paternal mitochondria (A) Time course of allophagy and embryogenesis previously reported.^8^^,^^28^ The general time course of early embryogenesis at ∼22 °C is shown in the frame. The post-fertilization cell cycle was classified into six phases based on morphological features identifiable by DIC imaging: phase 1, from sperm entry until the onset of eggshell formation; phase 2, from the onset of eggshell formation through meiosis II; phase 3, immediately after second polar body extrusion; phase 4, during pronuclear expansion; phase 5, during pronuclear migration; and phase 6, from the first mitosis to the two-cell stage. Images in Figures 4 and S4 were acquired according to these phase definitions. (B) Representative images of GFP::BCAS-3 and HSP-6::mCherry (paternal mitochondria) in zygotes at each phase defined in (A). Scale bars, 1 μm. (C) Quantification of GFP::BCAS-3 fluorescence intensity around paternal mitochondria at each phase. The GFP::BCAS-3 intensity around paternal mitochondria was normalized to the HSP-6::mCherry intensity in each paternal mitochondrion, and the mean normalized value in each zygote/embryo was plotted. 10 zygotes/embryos were analyzed at each phase. Error bars represent the mean ± SEM. (D) Representative images of GFP::BCAS-3 and mScarlet-I::LGG-1 in zygotes/embryos at each phase defined in (A). Scale bars, 1 μm. See also Figure S4.

We further evaluated the spatiotemporal dynamics of BCAS-3 localization during allophagy. We categorized the post-fertilization cell cycle into six phases based on morphological features of zygotes and embryos identifiable by differential interference contrast (DIC) imaging, and measured the morphological patterns and signal intensity of BCAS-3 in each phase (Figure 4A). In *C*. *elegans*, meiosis I and II are completed post-fertilization, and the first mitotic division occurs approximately 50 min after fertilization. In allophagy, the LGG-1-positive phagophore initially appears around paternal mitochondria as small puncta approximately 5–12 min after sperm–oocyte contact (late phase 1 ∼ early phase 2) and progressively encloses them from meiosis II to the pronuclear expansion stage (phases 2–5).^8^ In the pronuclear migration stage to the first mitosis stage (phases 5–6), these autophagosomes are transported to the lysosome-rich pericentrosomal region.^29^ Subsequently, sequestered paternal mitochondria are gradually degraded by the 16-cell stage.^1^ We observed minimal GFP::BCAS-3-positive puncta in early meiosis I (phase 1), followed by a sharp increase in puncta and cup- or ring-shaped structures through meiosis II (phases 2–3; Figures 4B and S4A). These signals subsequently declined during the pronuclear expansion and migration stages (phases 4–5) and were largely absent by first mitosis (phase 6; Figures 4B and S4A). Quantification of the signal intensity of GFP::BCAS-3 around paternal mitochondria confirmed that GFP::BCAS-3 recruitment reached its maximum during phases 2–3, followed by a gradual decline (Figures 4C and S4B). To elucidate the relationship with the phagophore membrane, we further compared GFP::BCAS-3 localization with that of mScarlet-I::LGG-1 (Figure 4D). During phases 2–3, GFP::BCAS-3 puncta and cup- or ring-shaped structures partially overlapped or were adjacent to mScarlet-I::LGG-1, representing growing phagophore membranes. During phases 4–5, when phagophores elongate and mature into autophagosomes, GFP::BCAS-3-positive structures decreased, and we observed small GFP::BCAS-3 foci associated with some of the mScarlet-I::LGG-1-positive rings. In phase 6, distinct GFP::BCAS-3 signals were no longer observed on most autophagosomes. The localization of PHAF-1::GFP around paternal mitochondria showed a temporal pattern similar to that of GFP::BCAS-3 (Figure S4C). These observations suggest that GFP::BCAS-3 and PHAF-1::GFP are enriched near the phagophore membrane specifically during the elongation phase.

### ATG-18 and EPG-6 are required at distinct steps of autophagosome formation from BCAS-3

The BCAS3 and WIPI families show structural similarity (Figure S1A).^15^ In human cells, BCAS3 is colocalized with WIPI1/2 and co-immunoprecipitated with WIPI1 under 0.1% paraformaldehyde treatment during Parkin-dependent mitophagy.^15^ These findings suggest that BCAS3 and WIPIs may cooperatively regulate autophagosome formation, although their precise functional relationships remain unclear. To investigate the role of BCAS-3 in allophagy in more detail, we examined its relationship with ATG-18 and EPG-6, which are closely related to human WIPI1/2 and WIPI3/4, respectively (Figure S1B)^14^ during this process.

**Figure 5:**
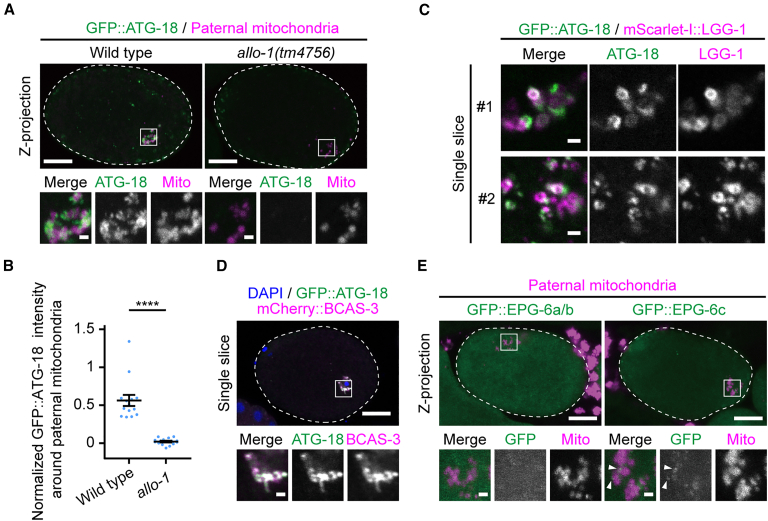
Localization of ATG-18 and EPG-6 during allophagy (A and B) ALLO-1-dependent recruitment of ATG-18 to paternal mitochondria. The GFP::ATG-18 intensity around paternal mitochondria was normalized to the HSP-6::mCherry (paternal mitochondria) intensity in each paternal mitochondrion, and the mean value for each zygote is shown. *n* = 14 (wild type) and 16 (*allo-1*) zygotes from late meiosis I to pronuclear expansion. Error bars represent the mean ± SEM. Statistical differences were determined using the Mann–Whitney U test. ∗∗∗∗*p* < 0.0001. (C) Partial colocalization of ATG-18 and LGG-1 during allophagy. (D) Colocalization of ATG-18 and BCAS-3 during allophagy. The signal from endogenous mCherry::BCAS-3 was detected using an anti-mCherry antibody. (E) EPG-6 localization during allophagy. The transgene GFP::EPG-6a/b potentially expresses both GFP::EPG-6a and GFP::EPG-6b. Arrowheads show GFP::EPG-6c foci around paternal mitochondria. At least 15 zygotes from late meiosis I to pronuclear expansion were observed, and representative images are shown (C–E). Dashed lines represent the outlines of the zygotes. Scale bars, 10 μm (upper panels in A, D, and E) and 1 μm (C and lower panels in A, D, and E). See also Figure S5.

First, we compared the localization of ATG-18 and EPG-6 with that of BCAS-3. Similar to BCAS-3, GFP::ATG-18 was localized to the punctate, cup- or ring-shaped structures around paternal mitochondria in an *allo-1*-dependent manner, and partially colocalized with mScarlet-I::LGG-1 (Figures 5A–5C and S5A). In addition, GFP::ATG-18 was colocalized with mCherry::BCAS-3, suggesting that they act together at the autophagosome formation site (Figure 5D). Simultaneous observation of GFP::ATG-18, mCherry::BCAS-3, and endogenous LGG-1 showed that the localizations of BCAS-3 and ATG-18 largely overlapped, whereas LGG-1 exhibited both overlapping and non-overlapping regions with BCAS-3 and ATG-18. This further suggests that BCAS-3 and ATG-18 are enriched in a specific region rather than being uniformly distributed across the entire phagophore membrane (Figure S5B). In contrast, EPG-6 exhibited a distinct localization pattern. *C*. *elegans* possesses four EPG-6 isoforms; among these, isoforms a, b, and c harbor the seven β-propeller structures, similar to ATG-18 and BCAS-3 (Figures S1A, S5C, and S5D). Of these, GFP::EPG-6c showed a weak association with paternal mitochondria, whereas GFP::EPG-6a/b, in which both isoforms are potentially expressed, showed a diffuse pattern (Figures 5E and S5E). This suggests that only a small portion of EPG-6c is recruited to paternal mitochondria compared to BCAS-3 and ATG-18, which is consistent with the less accumulation of EPG-6 at the autophagosome formation site during PGL-1 degradation.^14^

**Figure 6:**
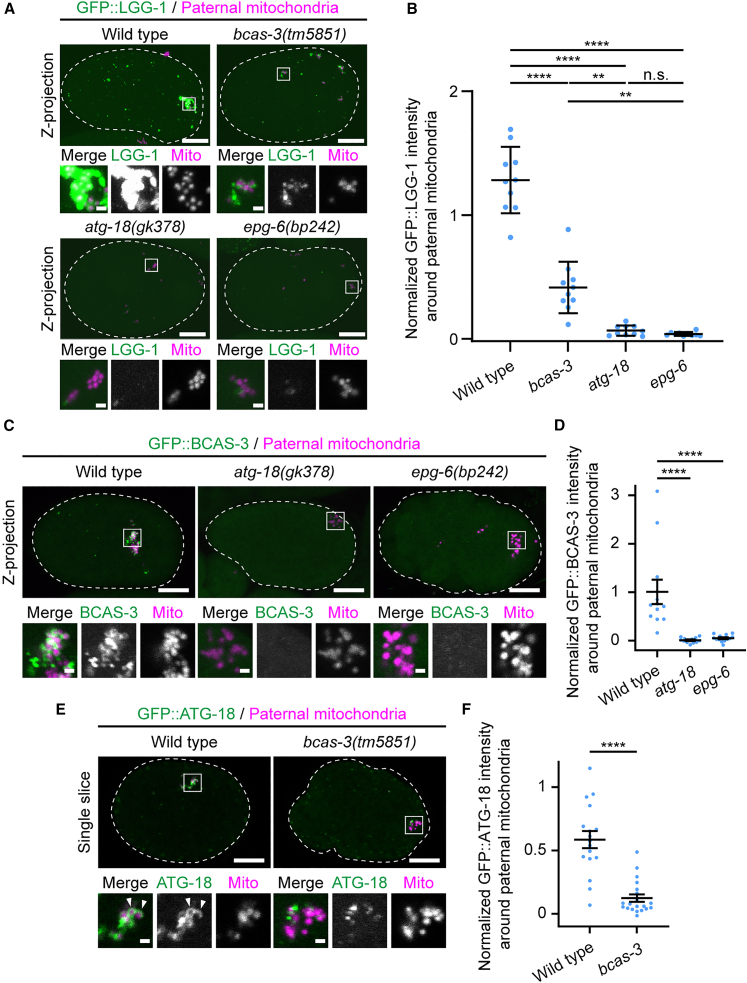
Functional relationship between BCAS-3 and WIPIs (A and B) Impaired autophagosome formation in the *bcas-3*, *atg-18,* and *epg-6* mutants. *n* = 10 zygotes per strain during pronuclear migration. (C and D) Impaired recruitment of BCAS-3 to paternal mitochondria in the *atg-18* and *epg-6* mutants. *n* = 12 (wild type) and 13 (*atg-18* and *epg-6*) zygotes from late meiosis I to pronuclear expansion. (E and F) Reduced accumulation of ATG-18 around paternal mitochondria in the *bcas-3* mutant. Arrowheads represent the elongated or cup-shaped structures. *n* = 17 (wild type) and 20 (*bcas-3*) zygotes from late meiosis I to pronuclear expansion. The GFP::LGG-1 (B), GFP::BCAS-3 (D), and GFP::ATG-18 (F) intensities around paternal mitochondria were normalized to the HSP-6::mCherry (paternal mitochondria) intensity in each paternal mitochondrion, and the mean value in each zygote is shown. Error bars represent the mean ± SEM. Statistical differences were determined using Welch’s one-way ANOVA followed by Games–Howell post hoc test (B), the Mann–Whitney U test followed by Holm correction (D) or the Mann–Whitney U test (F). n.s.: *p* > 0.05, ∗∗*p* < 0.01, and ∗∗∗∗*p* < 0.0001. Dashed lines represent the outlines of the zygotes. Scale bars, 10 μm (original images) and 1 μm (magnified images). See also Figure S6.

Next, we compared the phenotypes of the *bcas-3* with those of the *atg-18* and *epg-6* mutants. Similar to the *bcas-3* mutant, in *atg-18* and *epg-6* mutants, persistent paternal mitochondria were observed in embryos, suggesting that ATG-18 and EPG-6 are required for paternal mitochondrial degradation (Figures S6A and S6B). Nevertheless, the step impaired in autophagosome formation during allophagy differed from that observed in the *bcas-3* mutant. In the *bcas-3* mutant, GFP::LGG-1 was recruited to paternal mitochondria, but the fluorescent intensity was reduced compared to that in wild-type zygotes, and few GFP::LGG-1-positive structures fully sequestered paternal mitochondria. In contrast, in the *atg-18* and *epg-6* mutants, LGG-1 recruitment to paternal mitochondria was more severely impaired than in the *bcas-3* mutant (Figures 6A and 6B).

We also examined whether BCAS-3 recruitment is affected in *atg-18* and *epg-6* mutants. In wild-type zygotes, GFP::BCAS-3 was recruited to paternal mitochondria. However, this recruitment was not observed in the *atg-18* and *epg-6* mutants, suggesting that ATG-18 and EPG-6 are required for BCAS-3 recruitment to paternal mitochondria (Figures 6C and 6D). We confirmed that the expression levels of GFP::BCAS-3 remained largely unchanged in these mutants (Figure S6C). We also examined ATG-18 localization in the *bcas-3* mutant. Although GFP::ATG-18 was recruited to paternal mitochondria, the fluorescent intensity was significantly reduced in the *bcas-3* mutant (Figures 6E and 6F). This reduced recruitment of ATG-18 was not due to marked changes in the expression levels of ATG-18 in the *bcas-3* mutant (Figure S6D). These results suggest that ATG-18-dependent BCAS-3 recruitment promotes further ATG-18 accumulation around paternal mitochondria. We also found that the *phaf-1* mutant displayed similar defects in LGG-1 and ATG-18 recruitment to those seen in the *bcas-3* mutant, suggesting that these two proteins cooperate to facilitate proper phagophore formation (Figures S6E–S6I).

## Discussion

After fertilization, paternal mitochondria are recognized by the allophagy adaptor ALLO-1 and subsequently sequestered by the phagophore for degradation in the lysosome.^7^^,^^8^ Although we previously showed that ALLO-1 recruits the ULK1/Atg1 initiation complex, the detailed mechanisms underlying subsequent local autophagosome formation remain unclear. In this study, our genetic screen identified BCAS-3 and PHAF-1 as additional factors essential for allophagy.

Although BCAS3 and PHAF1 have been reported to be involved in autophagy, their precise roles remain to be elucidated. Our phenotypic analysis suggested that the phagophore is partially formed during allophagy, but further expansion is impaired in these mutants (Figures 1C and 6A). BCAS-3 accumulated around paternal mitochondria via its PI3P-binding site in the WD40 repeat domain (Figure 3E). BCAS-3 first appeared as puncta associated with LGG-1 during meiosis I. Subsequently, it showed a ring- or cup-shaped appearance that partially overlapped with the LGG-1-positive growing phagophore membrane, but these structures diminished and finally disappeared as autophagosome formation proceeded (Figures 4 and S4). Such temporal patterns may be reminiscent of those of Atg2, Atg9, and Atg18 in *Saccharomyces cerevisiae* and ATG2 in mammals, which localize to the edge of the growing phagophore membrane.^30^^,^^31^ It may also be similar to DFCP1-labeled omegasomes in mammals, PI3P-enriched ER subdomains associated with the phagophore membrane,^32^ although DFCP1 is not conserved in *C*. *elegans*. This pattern of BCAS-3 and PHAF-1 localization is consistent with the notion that they are directly involved in phagophore expansion.

Previous studies have shown that ATG-18 and EPG-6, *C*. *elegans* Atg18/WIPI homologs, regulate distinct steps of phagophore expansion during PGL-1 degradation. EPG-6 interacts with the lipid-transfer protein ATG-2 and is required for later steps of autophagosome formation than ATG-18.^14^^,^^33^^,^^34^ Although the role of ATG-18 in autophagosome formation remains unclear in *C*. *elegans*, one of the major functions of Atg18/WIPI family proteins in *S*. *cerevisiae* and mammals is to recruit Atg16/ATG16L1 to the phagophore, where the Atg16/ATG16L1 complex mediates the conjugation of ATG8 family proteins with phosphatidylethanolamine,^11^^,^^35^ implying that ATG-18 might recruit LGG-1 to the phagophore via ATG-16 in *C*. *elegans*. In allophagy, ATG-18, EPG-6, and BCAS-3 are all indispensable, and in *atg-18* or *epg-6* mutants, phagophore expansion was blocked at a very early stage (Figures 6A and 6B). Furthermore, BCAS-3 was hardly recruited to paternal mitochondria in the *atg-18* mutant, suggesting that BCAS-3 acts downstream of ATG-18 (Figures 6C and 6D). We also found that ATG-18 accumulation around paternal mitochondria was reduced in the *bcas-3* or *phaf-1* mutant, implying that the ATG-18-BCAS-3 feedback loop exists (Figures 6E and S6G). Such feedback mechanisms may facilitate the further accumulation of ATG-18, leading to phagophore expansion. Recently, Franco-Romero et al.^22^ showed that PHAF1 interacts with WIPI2, the ATG-18 homolog, and promotes its recruitment to the phagophore in mammals. Given that *C*. *elegans* BCAS-3 and PHAF-1 appear to function as a complex (Figure 1F), as in the case of human cells,^15^ BCAS-3 might mediate ATG-18 recruitment through its interaction with PHAF-1. Alternatively, BCAS-3 may have a distinct function that indirectly promotes ATG-18 recruitment. Although the hierarchy between WIPIs and BCAS3/PHAF1 during autophagosome formation remains unclear in mammals, the WIPI-BCAS3 feedback loop identified in allophagy may be conserved and important for autophagosome formation in mammals. BCAS3 has also been reported to interact with microtubules and intermediate filaments and regulate cytoskeletal organizations in mammals.^36^^,^^37^ However, no filamentous pattern of BCAS-3 was detected in *C*. *elegans* zygotes, and it remains unclear whether such a function is conserved in *C*. *elegans*. Further studies are necessary to elucidate the molecular functions of BCAS-3 and PHAF-1.

Although the loss of BCAS3 function leads to neurodevelopmental disorder,^17^^,^^18^^,^^19^ the underlying molecular mechanisms remain elusive. Our study revealed that BCAS-3 is involved in autophagy under physiological conditions and plays a crucial role in the progression of autophagosome formation during allophagy. In contrast, we found that BCAS-3 was less critical for PGL-1 autophagy (Figure 2C). These findings suggest that its requirement depends on the cargo or cellular context and that BCAS3 may contribute to neuronal function by regulating specific types of autophagy in humans. Since allophagy is highly dependent on BCAS-3 as well as ATG-18 and EPG-6, it provides a sensitive *in vivo* model for dissecting their functions in autophagy, potentially advancing our understanding of the mechanisms underlying human diseases associated with *BCAS3* mutations.

### Limitations of the study

In this study, we found that BCAS-3 is important for ATG-18 accumulation around paternal mitochondria (Figure 6E). However, we were unable to determine whether BCAS-3 also regulates EPG-6 localization because GFP::EPG-6c showed only weak accumulation around paternal mitochondrial membranes (Figure 5E). We also found that BCAS-3 and PHAF-1 are required for the degradation of paternal mitochondria and MOs, but are less important for PGL-1 degradation (Figure 2). The reason for this difference between the requirements of BCAS-3 and PHAF-1 remains unknown. Further comparative analyses of allophagy and PGL-1 degradation may provide insights into the roles of BCAS-3 and PHAF-1 in autophagosome formation. While the *bcas-3* and *phaf-1* mutants used in this study are fertile and do not exhibit obvious developmental defects under standard laboratory conditions, the *phaf-1* mutant has been reported to exhibit reduced lifespan.^22^ Identifying additional physiological phenotypes of these mutants remains an important subject for future investigation.

## Resource availability

### Lead contact

Requests for further information and resources should be directed to and will be fulfilled by the lead contact, Miyuki Sato (m-sato@gunma-u.ac.jp).

### Materials availability

The *C*. *elegans* strains and plasmids generated in this study are available from the lead contact upon reasonable request and after completing a material transfer agreement.

### Data and code availability

All data reported in this paper will be shared by the lead contact upon request. All commands and custom scripts for analyzing whole-genome sequencing are available as a Jupyter notebook on GitHub (https://github.com/YujiSue/Research/blob/main/NGSDataAnalysisColab/VariantDetection_step_by_step.ipynb). Any additional information required to reanalyze the data reported in this study is available from the lead contact upon request from the corresponding author.

## Acknowledgments

We thank Ken Sato, Naomi Terawaki, Mei Tajima, and other members of Sato’s laboratory for their technical support and discussions. We are grateful to Steven L’Hernault for supplying the MO antibody used in this study. Several strains were provided by the CGC, which is funded by the NIH Office of Research Infrastructure Programs (P40 OD010440) and the Japanese National Bioresource Project for the Experimental Animal “Nematode *C*. *elegans*.” This study was supported by 10.13039/501100001691JSPS 10.13039/501100001691KAKENHI (grant nos. 22KJ0444 to TN, 20J01777, 22K15097, and 25K18456 to TS, 21H02472 and 25K02268 to MS), 10.13039/501100001700MEXT KAKENHI (grant nos. 19H05712 and 24H02274 to MS, and 23H04923 to KY), 10.13039/100007449Takeda Science Foundation grant (to MS), 10.13039/100009619AMED-10.13039/501100003382CREST (grant no. JP20gm1410004 to NM), 10.13039/100009619AMED-PRIME (grant no. JP25gm6910030 to KY), The Cell Science Foundation (to KY) and the Joint Usage and Joint Research Program of IMCR, Gunma University, and Nanken-Kyoten, Science Tokyo.

## Author contributions

Conceptualization, T.N., W.K., K.Y., and M.S.; formal analysis, T.N. and Y.S.; funding acquisition, T.N., T.S., and M.S.; investigation, T.N. and T.S.; software, Y.S.; supervision, S.M., K.Y., N.M., and M.S.; writing, T.N., T.S., and M.S.

## Declaration of interests

The authors declare that they have no conflict of interest.

## STAR★Methods

### Key resources table

**Table:** 

REAGENT or RESOURCE	SOURCE	IDENTIFIER
**Antibodies**		

Mouse monoclonal anti-MO antibody	Okamoto and Thomson^24^	N/A
Rat monoclonal anti-mCherry antibody	Thermo Fisher Scientific	Cat# M11217, RRID: AB_2536611
Rabbit polyclonal anti-LGG-1 antibody	Sato and Sato^1^	N/A
Goat polyclonal anti-GFP antibody	Fitzgerald Industries International	Cat# 70R-GG001, RRID: AB_1286216
Mouse monoclonal anti-beta-actin antibody	Santa Cruz Biotechnology	Cat# sc-47778, RRID: AB_626632
Goat anti-Mouse IgG (H + L) Cross-Adsorbed Secondary Antibody, Alexa Fluor™ 555	Thermo Fisher Scientific	Cat# A-21422, RRID: AB_2535844
Goat anti-Rat IgG (H + L) Cross-Adsorbed Secondary Antibody, Alexa Fluor™ 594	Thermo Fisher Scientific	Cat# A-11007, RRID: AB_10561522
Goat anti-Rabbit IgG (H + L) Highly Cross-Adsorbed Secondary Antibody, Alexa Fluor™ Plus 647	Thermo Fisher Scientific	Cat# A32733, RRID: AB_2866492
Peroxidase-conjugated donkey anti-goat immunoglobulin G antibody	Merck	Cat# AP180P, RRID: RRID: AB_92573
Peroxidase-conjugated goat anti-mouse immunoglobulin G antibody	Jackson ImmunoResearch	Cat# 115-035-003, RRID: AB_10015289

**Bacterial and virus strains**		

*E*. *coli* OP50	Caenorhabditis Genetic Center (CGC)	RRID: WB-STRAIN: WBStrain00041969
*E*. *coli* HB101	CGC	RRID: WB-STRAIN: WBStrain00041075

**Chemicals, peptides, and recombinant proteins**		

Ethyl methanesulfonate (EMS)	Sigma–Aldrich	Cat# M0880
SuperSignal™ West Pico PLUS Chemiluminescent Substrate	Thermo Fisher Scientific	Cat# 34580

**Experimental models: Organisms/strains**		

*C*. *elegans*: Wild-type Bristol (N2) strain	CGC	N2
*C*. *elegans*: *allo-1(tm4756)*	Sato et al.^7^	GK1585
*C*. *elegans*: *bcas-3(tm5851)*	Japanese National Bioresource Project	GK2467
*C*. *elegans*: *phaf-1(dk3)*	This paper	GK2678
*C*. *elegans*: *dkIs970[pie-1p*:*:mScarlet-I*:*:lgg-1]*; *dkIs623[spe-11p*:*:hsp-6*:*:gfp]*	This paper	GK2140
*C*. *elegans*: *bcas-3(dk1)*; *dkIs970[pie-1p*:*: mScarlet-I*:*:lgg-1]*; *dkIs623[spe-11p*:*:hsp-6*:*:gfp]*	This paper	GK2656
*C*. *elegans*: *phaf-1(dk2)*; *dkIs970[pie-1p*:*: mScarlet-I*:*:lgg-1]*; *dkIs623[spe-11p*:*:hsp-6*:*:gfp]*	This paper	GK2655
*C*. *elegans*: *bcas-3(tm5851)*; *dkIs970[pie-1p*:*: mScarlet-I*:*:lgg-1]*; *dkIs623[spe-11p*:*:hsp-6*:*:gfp]*	This paper	GK2760
*C*. *elegans*: *phaf-1(dk3)*; *dkIs970[pie-1p*:*: mScarlet-I*:*:lgg-1]*; *dkIs623[spe-11p*:*:hsp-6*:*:gfp]*	This paper	GK2761
*C*. *elegans*: *him-5(e1490)*; *cdIs5[myo-3p*:*:ssDsRed]*	This paper	GK2492
*C*. *elegans*: *bcas-3(tm5851)*; *him-5(e1490)*; *cdIs5[myo-3p*:*:ssDsRed]*	This paper	GK2713
*C*. *elegans*: *phaf-1(dk3)*; *him-5(e1490)*; *cdIs5[myo-3p*:*:ssDsRed]*	This paper	GK2674
*C*. *elegans*: *pgl-1(ax3122[pgl-1*:*:gfp])*	CGC	JH3269
*C*. *elegans*: *epg-6(bp242)*; *pgl-1(ax3122[pgl-1*:*:gfp])*	This paper	GK2788
*C*. *elegans*: *bcas-3(tm5851)*; *pgl-1(ax3122[pgl-1*:*:gfp])*	This paper	GK2801
*C*. *elegans*: *phaf-1(dk3)*; *pgl-1(ax3122[pgl-1*:*: gfp])*	This paper	GK2800
*C*. *elegans*: *dkIs698[spe-11p*:*:hsp-6*:*:mcherry]*; *dkIs1069[pie-1p*:*:gfp*:*:bcas-3]*	This paper	GK2428
*C*. *elegans*: *dkIs1069[pie-1p*:*:gfp*:*:bcas-3]*; *dkIs970[pie-1p*:*:mScarlet-I*:*:lgg-1]*	This paper	GK2426
*C*. *elegans*: *dkIs698[spe-11p*:*:hsp-6*:*:mcherry]*; *dkIs1198[pie-1p*:*:phaf-1*:*:gfp]*	This paper	GK2682
*C*. *elegans*: *dkIs1198[pie-1p*:*:phaf-1*:*:gfp]*; *dkIs970[pie-1p*:*:mScarlet-I*:*:lgg-1]*	This paper	GK2990
*C*. *elegans*: *bcas-3(tm5851)*; *dkIs698[spe-11p*:*:hsp-6*:*:mcherry]*	This paper	GK2468
*C*. *elegans*: *phaf-1(dk3)*; *dkIs698[spe-11p*:*:hsp-6*:*:mcherry]*	This paper	GK2703
*C*. *elegans*: *bcas-3(tm5851)*; *dkIs698[spe-11p*:*:hsp-6*:*:mcherry]*; *dkIs1069[pie-1p*:*:gfp*:*:bcas-3]*	This paper	GK2469
*C*. *elegans*: *phaf-1(dk3)*; *dkIs698[spe-11p*:*:hsp-6*:*:mcherry]*; *dkIs1198[pie-1p*:*:phaf-1*:*:gfp]*	This paper	GK2961
*C*. *elegans*: *bcas-3(tm5851)*; *dkIs698[spe-11p*:*: hsp-6*:*:mcherry]*; *dkIs1198[pie-1p*:*:phaf-1*:*:gfp]*	This paper	GK2706
*C*. *elegans*: *phaf-1(dk3)*; *dkIs698[spe-11p*:*: hsp-6*:*:mcherry]*; *dkIs1069[pie-1p*:*:gfp*:*:bcas-3]*	This paper	GK2725
*C*. *elegans*: *bcas-3(tm5851)*; *dkIs698[spe-11p*:*: hsp-6*:*:mcherry]*; *dkIs1233[pie-1p*:*:gfp*:*:bcas-3 H567A]*	This paper	GK2943
*C*. *elegans*: *allo-1(tm4756)*; *dkIs698[spe-11p*: :*hsp-6*:*:mcherry]*; *dkIs1069[pie-1p*:*:gfp*:*:bcas-3]*	This paper	GK2429
*C*. *elegans*: *allo-1(tm4756)*; *dkIs698[spe-11p*:*: hsp-6*:*:mcherry]*; *dkIs1198[pie-1p*:*:phaf-1*:*:gfp]*	This paper	GK2705
*C*. *elegans*: *dkIs623[spe-11p*:*:hsp-6*:*:gfp]*; *bcas-3(syb10258[mcherry*:*:bcas-3])*	This paper	GK2904
*C*. *elegans*: *allo-1(tm4756)*; *dkIs623[spe-11p*:*: hsp-6*:*:gfp]*; *bcas-3(syb10258[mcherry*:*:bcas-3])*	This paper	GK2905
*C*. *elegans*: *dkIs698[spe-11p*:*:hsp-6*:*:mcherry]*; *dkIs743[pie-1p*:*:gfp*:*:atg-18]*	This paper	GK2771
*C*. *elegans*: *allo-1(tm4756)*; *dkIs698[spe-11p*:*: hsp-6*:*:mcherry]*; *dkIs743[pie-1p*:*:gfp*:*:atg-18]*	This paper	GK2772
*C*. *elegans*: *dkIs743[pie-1p*:*:gfp*:*:atg-18]*; *dkIs970[pie-1p*:*:mScarlet-I*:*:lgg-1]*	This paper	GK2787
*C*. *elegans*: *dkIs743[pie-1p*:*:gfp*:*:atg-18]*; *bcas-3(syb10258[mcherry*:*:bcas-3])*	This paper	GK2855
*C*. *elegans*: *dkIs698[spe-11p*:*:hsp-6*:*:mcherry]*; *dkIs1192[pie-1p*:*:gfp*:*:epg-6a/b]*	This paper	GK2720
*C*. *elegans*: *dkIs698[spe-11p*:*:hsp-6*:*:mcherry]*; *dkIs1201[pie-1p*:*:gfp*:*:epg-6c]*	This paper	GK2721
*C*. *elegans*: *dkIs698[spe-11p*:*:hsp-6*:*:mcherry]*; *dkIs398[pie-1p*:*:gfp*:*:lgg-1]*	Sato et al.^7^	GK1370
*C*. *elegans*: *bcas-3(tm5851)*; *dkIs698[spe-11p*:*: hsp-6*:*:mcherry]*; *dkIs398[pie-1p*:*:gfp*:*:lgg-1]*	This paper	GK2248
*C*. *elegans*: *atg-18(gk378)*; *dkIs698[spe-11p*:*: hsp-6*:*:mcherry]*; *dkIs398[pie-1p*:*:gfp*:*:lgg-1]*	This paper	GK2624
*C*. *elegans*: *epg-6(bp242)*; *dkIs698[spe-11p*:*: hsp-6*:*:mcherry]*; *dkIs398[pie-1p*:*:gfp*:*:lgg-1]*	This paper	GK2611
*C*. *elegans*: *dkIs699[spe-11p*:*:hsp-6*:*:mcherry]*; *dkIs399[pie-1p*:*:gfp*:*:lgg-1]*	This paper	GK2015
*C*. *elegans*: *dkIs699[spe-11p*:*:hsp-6*:*:mcherry]*; *dkIs399[pie-1p*:*:gfp*:*:lgg-1]*; *phaf-1(dk3)*	This paper	GK2726
*C*. *elegans*: *atg-18(gk378)*; *dkIs698[spe-11p*:*: hsp-6*:*:mcherry]*; *dkIs1069[pie-1p*:*:gfp*:*:bcas-3]*	This paper	GK2950
*C*. *elegans*: *epg-6(bp242)*; *dkIs698[spe-11p*:*: hsp-6*:*:mcherry]*; *dkIs1069[pie-1p*:*:gfp*:*:bcas-3]*	This paper	GK2708
*C*. *elegans*: *bcas-3(tm5851)*; *dkIs698[spe-11p*:*: hsp-6*:*:mcherry]*; *dkIs743[pie-1p*:*:gfp*:*:atg-18]*	This paper	GK2804
*C*. *elegans*: *phaf-1(dk3)*; *dkIs698[spe-11p*:*: hsp-6*:*:mcherry]*; *dkIs743[pie-1p*:*:gfp*:*:atg-18]*	This paper	GK3070
*S*. *cerevisiae*: *MATα trp1 his3 ura3 leu2*:*:6 LexAop-LEU2*	OriGene Technologies	EGY48

**Oligonucleotides**		

See Table S1	–	N/A

**Recombinant DNA**		

pID2.02 *pie-1p*:*:mScarlet-I*:*:lgg-1*	This paper	N/A
pID3.01B *pie-1p*:*:gfp*:*:bcas-3*	This paper	N/A
pID3.01B *pie-1p*:*:gfp*:*:atg-18*	This paper	N/A
pID3.01B *pie-1p*:*:gfp*:*:epg-6a/b*	This paper	N/A
pID3.01B *pie-1p*:*:gfp*:*:epg-6c*	This paper	N/A
pID3.01B *pie-1p*:*:gfp*:*:bcas-3 H567A*	This paper	N/A
pID2.02 *pie-1p*:*:phaf-1*:*:gfp*	This paper	N/A
pJG4-5 (prey vector)	OriGene Technologies	N/A
pJG4-5 *phaf-1b*	This paper	N/A
pJG4-5 *phaf-1b G307R*	This paper	N/A
pEG202 (bait vector)	OriGene Technologies	N/A
pEG202 *bcas-3b*	This paper	N/A
pMLS134 *phaf-1*	This paper	N/A
pID3.01B *pie-1p*:*:gfp*:*:lgg-1*	Sato and Sato^1^	N/A
pMS20.2 *spe-11p*:*:hsp-6*:*:m*c*herry*	Sato et al.^7^	N/A
*eft-3p*:*:cas9-SV40_NLS*:*:tbb-2 3′UTR*	Friedland et al.^38^	Addgene #46168

**Software and algorithms**		

E-CRISP	Heigwer et al.^39^	http://www.e-crisp.org/
PrimerQuest™ program	Integrated DNA Technologies	https://sg.idtdna.com/pages/tools/primerquest
R (version: 4.5.1)	The R project	https://www.r-project.org/
GraphPad Prism 11	GraphPad Software	https://www.graphpad.com/
Fiji (ImageJ version 1.54p)	National Institute of Health	https://imagej.net/software/fiji/
FastQC (version: 0.12.1)	Andrews^40^	http://www.bioinformatics.babraham.ac.uk/projects/fastqc/
Fastp (version: 0.23.2)	Chen et al.^41^	https://github.com/OpenGene/fastp
BWA-MEM (version: 0.7.17)	Li^42^	https://github.com/lh3/bwa
Samtools (version: 1.19.2)	Li et al.^43^	https://www.htslib.org/
Picard (version: 2.27.5)	Broad Institute	http://broadinstitute.github.io/picard/
GATK (version: 4.5.0.0)	Van der Auwera et al.^44^	https://gatk.broadinstitute.org/hc/en-us
SIFT-4G (version: MO-SIFT-21Apr14)	Vaser et al.^45^	https://sift.bii.a-star.edu.sg/sift4g/
Sutoku (version 1.2.1)	Suehiro et al.^46^	https://github.com/YujiSue/Sutoku
Clustal X 2.1	Larkin et al.^47^	http://www.clustal.org/clustal2/
AlphaFold server	Abramson et al.^48^	https://alphafoldserver.com/
ChimeraX (version 1.10.1)	Goddard et al.^49^; Pettersen et al.^50^; Meng et al.^51^	https://www.rbvi.ucsf.edu/chimerax/
Gblocks (version 0.91.1)	Castresana^52^; Talavera and Castresana^53^; Lemoine et al.^54^	https://ngphylogeny.fr/tools/tool/276/form
PhyML 3.0	Guindon et al.^55^	http://www.atgc-montpellier.fr/phyml/
Commands and custom scripts for analyzing whole-genome sequencing	This paper	https://github.com/YujiSue/Research/blob/main/NGSDataAnalysisColab/VariantDetection_step_by_step.ipynb

### Experimental model and study participant details

*Caenorhabditis elegans* was maintained at 20°C or 25°C on the nematode growth medium (NGM) plate (3 g NaCl, 2.5 g peptone, 17 g agar, 1 mL of 1 M CaCl_2_, 1 mL of 1 M MgSO_4_, 5 mg cholesterol, 25 mL of 1 M KPO_4_ buffer pH 6.0, and up to 1 L distilled water) seeded with the *Escherichia* *coli* strain OP50, as previously described.^56^ *The unc-119(ed3)* mutant was maintained on the NGM-lite plate (2 g NaCl, 4 g tryptone, 3 g KH_2_PO_4_, 0.5 g K_2_HPO_4_, 8 mg cholesterol, 20 g agar, 100 mg streptomycin, 1 mL of 1 M CaCl_2_, and up to 1 L distilled water) seeded with the *E*. *coli* strain HB101 at 20°C.

### Method details

#### Vector construction

The primers used in this study are listed in Table S1.

To construct the vector carrying *pie-1p*:*:mScarlet-I*:*:lgg-1*, a genomic fragment of *lgg-1* and a fragment encoding *mScarlet-I* were amplified by PCR using the N2 genome and pMS050 (Addgene #91826)^57^ as templates, respectively. A fragment of the vector containing the *pie-1* promoter and terminator was amplified using PCR with pID2.02 (ref.^58^) as the template. The fragments of *lgg-1* and *mScarlet-I* were subcloned into the pID2.02 vector using InFusion Cloning Kit (Clontech).

To construct vectors carrying *pie-1p*:*:gfp*:*:bcas-3*, *pie-1p*:*:gfp*:*:atg-18*, *pie-1p*:*:gfp*:*:epg-6a/b*, and *pie-1p*:*:gfp*:*:epg-6c*, genomic fragments of *F56C9*.*10/bcas-3*, *atg-18*, *epg-6a/b*, *and epg-6c* were amplified by PCR using the N2 genome as a template and cloned into pDONR221 using Gateway Recombination with a BP clonase (Thermo Fisher Scientific). These fragments were subcloned into pID3.01B^59^ to express N-terminal GFP fusions under the *pie-1* promoter using Gateway Recombination using LR clonase (Thermo Fisher Scientific). The H567A mutation was introduced into pDONR221 *bcas-3* by PCR, and then the *bcas-3b H567A* fragment was subcloned into pID3.01B using LR clonase.

To construct the vector carrying *pie-1p*:*:phaf-1*:*:gfp*, genomic fragments covering the 1st-to-9th exons and 10th-to-12th exons of the *phaf-1* gene were amplified by PCR using the N2 genome as a template and fused by the 2nd PCR, because the *phaf-1* gene has a large 9th intron that contains another gene. The resulting *phaf-1* genomic fragment without the 9th intron was cloned into pDONR221 using BP clonase (pDONR221 *phaf-1*). A fragment of *phaf-1* without the 9th intron and a fragment of the vector containing the *pie-1* promoter, terminator, and *gfp* was amplified using PCR, using pDONR221 *phaf-1* and pID2.02 *pie-1p*:*:epg-7*:*:gfp*^8^ as the template, respectively. The *phaf-1* fragment was cloned into pID2.02 using InFusion Cloning Kit.

To construct the vectors used in a yeast two-hybrid analysis, coding sequences of isoform b of *bcas-3* and *phaf-1* were amplified by PCR from the cDNA library of the DupLEX-A Yeast Two-Hybrid System (OriGene Technologies) and cloned into pDONR221 using BP clonase. These fragments were subcloned into pEG202 (bait vector) and pJG4-5 (prey vector) harboring a Gateway cassette^60^ using LR clonase, respectively. G307R mutation was introduced into pDONR221 *phaf-1b* by PCR, and then the *phaf-1b G307R* fragment was subcloned into pJG4-5 using LR clonase.

To construct the vectors expressing sgRNA for creating *phaf-1(dk3)* by CRISPR/Cas9, we used SapTrap reaction.^61^ Briefly, complementary oligos were diluted to 10 μM each in 1× oligo annealing buffer (20 mM Tris-Cl, pH 7.5; 50 mM NaCl; 1 mM MgCl_2_), heated to 95°C in a heat block, and gradually cooled to room temperature over 1 h. For sgRNA assembly, equal volumes of 50 nM pMLS134 (Addgene #73714)^61^ and the annealed oligo mixture were mixed. Subsequently, 0.5 μL of this DNA mixture was combined with 2 μL of a SapTrap enzyme mix consisting of 5 μL of 10× CutSmart buffer (New England Biolabs), 22.5 μL of distilled water, 5 μL of 10 mM ATP, 0.25 μL of 1 M DTT, 1.25 μL of T4 DNA ligase (400 U/μL), 1 μL of T4 polynucleotide kinase (10 U/μL), and 5 μL of SapI (10 U/μL). Reactions were incubated at 20°C–25°C overnight, after which T4 DNA ligase was inactivated at 65°C for 30 min. Following inactivation, 2.5 μL of 1× CutSmart buffer containing 2 U/μL of BamHI was added, and the mixture was incubated at 37°C for 1 h. 2 μL of the reaction was used for transformation. gRNA targeting sites were designed using E-CRISP^39^ and PrimerQuest program (Integrated DNA Technologies).

#### *C. elegans* strain

The strains used in this study are listed in key resources table. The *unc-119(ed3)*, *atg-18(gk378)*, *epg-6(bp242)*, and *him-5(e1490)* mutants and *pgl-1(ax3122[pgl-1*:*:gfp])* were obtained from the Caenorhabditis Genetic Center. The *allo-1(tm4756)* and *bcas-3(tm5851)* alleles were provided by the Japanese National Bioresource Project for the Experimental Animal ‘Nematode *C*. *elegans*’. The *allo-1(tm4756)*, *bcas-3(tm5851)*, and *atg-18(gk378)* mutants were backcrossed with wild-type N2 at least once.

#### Generation of transgenic *C. elegans*

Transgenic lines expressing fluorescent protein-tagged proteins were generated using microparticle bombardment as previously described.^23^^,^^62^ Briefly, starved L1 larvae of the *unc-119(ed3)* mutant were incubated on NGM-lite plates seeded with the *E*. *coli* strain HB101 and chicken egg solution for 4 days at 20°C. The plasmid DNA was coated onto gold microcarriers (Bio-Rad) using CaCl_2_ and spermidine and introduced into the *unc-119(ed3)* mutant using the PDS-1000/He particle delivery system (Bio-Rad). The transgenic *C*. *elegans* were incubated on the NGM-lite plate with HB101 at 25°C for more than 2 weeks, and non-*unc (uncoordinated)* nematodes were transferred onto the NGM plate seeded with OP50.

The transgenic lines used in this study included *dkIs623[spe-11p*:*:hsp-6*:*:gfp]*,^1^ *dkIs398[pie-1p*:*:gfp*:*:lgg-1]*,^1^ *dkIs698[spe-11p*:*:hsp-6*:*:mcherry]*,^7^ and *cdIs5[myo-3p*:*:ssDsRed]*.^63^

To generate the *phaf-1(dk3)* mutant using the CRISPR-Cas9 system, we used a co-conversion strategy with *dpy-10(cn64)* as a marker, following the protocol described by Arribere et al.^64^ Young adult N2 hermaphrodites were microinjected in the gonad with an injection mixture containing 50 ng/μL sgRNA vectors, 50 ng/μL repair templates for *phaf-1* and *dpy-10(cn64)* (Integrated DNA Technologies), and 50 ng/μL Cas9 plasmid (*eft-3p*:*:cas9-SV40_NLS*:*:tbb-2 3′UTR*, Addgene #46168).^38^ F1 progeny exhibiting the roller phenotype were singled out, and the genotype of *phaf-1* was determined by single-worm PCR. Subsequently, an F2 animal carrying a deletion in *phaf-1* but lacking the *dpy-10(cn64)* phenotype was identified and established as the mutant line. In this mutant, the entire 5th and 6th exons and a part of the 7th exon, which correspond to P166 to I241, were deleted.

To generate the strain expressing mCherry::BCAS-3, a DNA fragment encoding mCherry (dpiRNA construct) was inserted after the start codon of the *bcas-3* gene using the CRISPR-Cas9 system by SUNY Biotech.

#### Genetic screening

Larvae of the strain GK2140 [*spe-11p*:*:hsp-6*:*:gfp*; *pie-1p*:*:mScarlet-I*:*:lgg-1*] were collected from L4-larvae rich an NGM plate with M9 buffer (3 g KH_2_PO_4_, 6 g Na_2_HPO_4_, 5 g NaCl, 1 mL 1 M MgSO_4_, and up to 1 L distilled water), washed with M9 and incubated in the M9 buffer containing 100-fold dilution of ethyl methanesulfonate (EMS; Cat# M0880, Sigma–Aldrich) at 20°C for 4 h. After washing with the M9 buffer, an L4 larva (P0) was singled out to an NGM plate seeded with OP50. Three to five progenies (F1) from EMS-mutagenized nematodes were placed together on a single NGM plate seeded with OP50 (at most five plates per P0 nematode), and up to eight progenies (F2) from each plate were singled out to individual NGM plates seeded with OP50. Among more than 10,000 plates where F2 progeny were singled out, approximately 60% of them were examined by confocal microscopy, due to the absence of gravid hermaphrodite on the remaining plates.

For screening of mutants defective in paternal mitochondrial degradation, we first isolated mutants in which paternal mitochondria persisted during embryogenesis. Gravid hermaphrodites were placed in M9 buffer containing 20 mM levamisole on 1.5% (w/v) agar pad and observed using a confocal laser-scanning microscope FV1000 (Evident) with UPlanSApo 60×/1.35 numerical aperture (NA) oil-immersion objective lens (Evident). To determine which steps of allophagy are impaired in these mutants, we examined the localization of mScarlet-I::LGG-1 in zygotes. Gravid hermaphrodites were dissected in M9 buffer containing 20 mM levamisole on a coverslip and mounted on a 1.5% (w/v) agar pad on a glass slide. Zygotes were observed using FV1000. To examine whether mutants isolated in this screening were alleles of allophagy-defective mutants previously reported,^7^^,^^8^ we crossed isolated mutants with allophagy-defective mutants, such as *allo-1* mutants, and examined whether allophagy defects were recovered in their progeny.

For the whole-genome sequence, *dk1* and *dk2* mutants were backcrossed with the parental strain GK2140 five times. Starved L1 larvae in GK2140 and these mutants were collected with M9 buffer and frozen at −80°C for at least overnight. Genomic DNA was obtained from these samples using DNeasy blood and tissue kits for DNA isolation (Qiagen) according to the manufacturer’s instructions. Short-read DNA sequencing was carried out on the Illumina NovaSeq X Plus by Eurofins Genomics K.K. using 150-bp paired-end sequencing. To identify the genes responsible for defective allophagy in *dk1* and *dk2* mutants, whole-genome sequencing data, provided as paired-end FASTQ files, were first subjected to quality control and trimming. Initial quality assessment of the sequence data was conducted with the FastQC tool (version 0.12.1; http://www.bioinformatics.babraham.ac.uk/projects/fastqc/).^40^ The remaining adapter sequences and low-quality reads were subsequently removed using fastp (version 0.23.2),^41^ a process that ensured the integrity and quality of the subsequent data for downstream analysis. The post-filter quality was confirmed again with the FastQC tool. The processed, high-quality reads were mapped to the *C*. *elegans* reference genome (WBCel235). Reads were then aligned to the indexed reference using BWA-MEM (version 0.7.17),^42^ converted to BAM format, sorted, and indexed using samtools (version 1.19.2).^43^ Potential PCR duplicates were marked using Picard (version 2.27.5; http://broadinstitute.github.io/picard/). Single-nucleotide variants and small insertions/deletions were called using GATK (version 4.5.0.0)^44^ HaplotypeCaller in GVCF mode, followed by GenotypeGVCFs to produce a final VCF file for each sample. The functional impact of the identified variants was then predicted using SIFT4G (version MO-SIFT-21Apr14).^45^ The SIFT4G_Annotator was run against the *C*. *elegans* WBcel235.83 database to classify variants based on their predicted effect. Structural variants (SVs) were detected using the proprietary tool Sutoku (version 1.2.1).^46^ To prioritize candidate causal mutations, an additional filter was applied to the annotated variants using the custom script. Specifically, variants were filtered to select homozygous variants and G:C to A:T transitions, known as typical variants induced by EMS mutagenesis. The process was executed on Google Colab. All commands and custom scripts are available as a Jupyter notebook on GitHub (https://github.com/YujiSue/Research/blob/main/NGSDataAnalysisColab/VariantDetection_step_by_step.ipynb).

We examined whether any of the identified mutations were present in genes involved in autophagy. We found a nonsense mutation in *bcas-3* and a nonsynonymous mutation in *phaf-1*. To confirm that mutations in these genes are responsible for defects in allophagy, we crossed *dk1* and *dk2* mutants with males of *bcas-3(tm5851)* or *phaf-1(dk3)* mutants, respectively, and examined whether allophagy defects were recovered in their progeny. For this complementation assay, nematodes carrying the *him-5(e1490)*; *cdIs5[myo-3p*:*:ssDsRed]* background were used to increase the frequency of males and to verify successful crosses.

#### Confocal microscope observation

To obtain images of live zygotes and embryos, nematodes were grown to the L4 stage at either 20°C or 25°C on NGM plates seeded with OP50 and then incubated at 20°C for 24–48 h before dissection. Adult hermaphrodites were dissected in M9 buffer containing 20 mM levamisole on a coverslip using syringe needles. The samples were then mounted on a 1.5% (w/v) agar pad on a glass slide. Zygotes and embryos were observed using a confocal laser-scanning microscope FV1200 (Evident) with a UPlanSApo 60×/1.4 NA oil-immersion objective lens (Evident) or FV4000 (Evident) with UPlanXApo 60×/1.42 NA oil-immersion objective lens (Evident) or UPlanXApo 100×/1.45 NA oil-immersion objective lens (Evident).

To observe PGL-1::GFP in embryos, gravid hermaphrodites grown on NGM plates seeded with OP50 at 20°C were transferred to NGM plates without *E*. *coli* for 4 h to keep later-stage embryos in the uterus and dissected in M9 buffer containing 20 mM levamisole on a coverslip using syringe needles, mounted on an adhesive glass slide (MAS-coated glass slide; Matsunami), and frozen at −80°C for at least overnight. After removing the coverslip, the sample was sequentially fixed in methanol for 5 min and in acetone for 2 min at −20°C. Fixed samples were blocked with PTB buffer (phosphate-buffered saline [PBS] buffer containing 1% [w/v] bovine serum albumin [BSA], 0.1% [v/v] Tween 20, 0.05% [w/v] NaN_3_ and 1 mM EDTA) for 1 h and mounted with SlowFade Diamond (Thermo Fisher Scientific) with 4′,6-diamidino-2-phenylindole (DAPI). Embryos were observed using FV4000.

Observation of MOs were performed as previously described.^8^^,^^23^ Gravid hermaphrodites grown on NGM plates seeded with OP50 at 20°C were transferred onto the NGM plate without *E*. *coli* to remove excess *E*. *coli*. Embryos were fixed with methanol and acetone, as described above. Fixed embryos were blocked with PTB buffer for 1 h and incubated with anti-MO antibody (1CB4 [ref.^24^]; dilution 1:250) in PTB buffer at 4°C overnight. The samples were washed with PTC buffer (PBS buffer containing 0.1% [w/v] BSA, 0.1% [v/v] Tween 20, 0.05% [w/v] NaN_3_ and 1 mM EDTA) nine times, and incubated with Goat anti-Mouse IgG (H + L) Cross-Adsorbed Secondary Antibody, Alexa Fluor 555 (dilution 1:1000; Cat# A-21422, RRID: AB_2535844, Thermo Fisher Scientific) in PTB buffer at 25°C for more than 3 h. The samples were washed nine times with PTC buffer, mounted with SlowFade Diamond with DAPI and observed using FV4000.

To examine localization of mCherry::BCAS-3, L4 larvae were incubated on NGM plates seeded with OP50 at 20°C for 24–48 h before dissection. The adult hermaphrodites were transferred onto the NGM plates without *E*. *coli*, and dissected embryos were frozen at −80°C at least overnight, as described above. Zygotes were first immersed in methanol at −20 °C for 15 s and then fixed in the fixative solution (3.2% [w/v] paraformaldehyde, 0.24 M sorbitol, 100 mM PIPES, 5 mM EDTA, and 5 mM MgCl_2_, pH 6.9). After washing with PEMT buffer (0.1% [v/v] Triton X-100, 100 mM PIPES, 5 mM EDTA, and 5 mM MgCl_2_, pH 6.9) nine times, fixed zygotes were blocked with PEMTB buffer (PEMT buffer containing 0.1% [w/v] BSA) for 30 min and then incubated with the primary antibody in PEMTB buffer at 4°C overnight. After washing with PEMT buffer nine times, the samples were incubated with the secondary antibody in PEMTB buffer at 25°C for more than 3 h. After washing with PEMT buffer nine times, the samples were mounted with SlowFade Diamond with DAPI and observed using FV4000. The primary antibodies used in immunostaining with paraformaldehyde fixation were rabbit polyclonal anti-LGG-1 antibody (dilution 1:800)^1^ and/or rat monoclonal anti-mCherry antibody (dilution 1:100; Cat# M11217, RRID: AB_2536611, Thermo Fisher Scientific). The secondary antibodies used in immunostaining with paraformaldehyde fixation were Goat anti-Rat IgG (H + L) Cross-Adsorbed Secondary Antibody, Alexa Fluor 594 (dilution 1:1000; Cat# A-11007, RRID: AB_10561522, Thermo Fisher Scientific) and/or Goat anti-Mouse IgG (H + L) Highly Cross-Adsorbed Secondary Antibody, Alexa Fluor Plus 647 (dilution 1:1000; Cat# A32733, RRID: AB_2866492, Thermo Fisher Scientific).

To obtain Z-projection images, zygotes and embryos were scanned at 1 μm intervals, and 11 z stack images (10 μm thick) were projected with maximum intensity projection using Fiji software (ImageJ version 1.54p).^65^

#### Yeast two-hybrid analysis

The prey and bait vectors were transformed into *S*. *cerevisiae* strain EGY48 carrying the reporter vector pSH18-34, as described previously.^60^ Yeast carrying the pJG4-5 and pEG202 vectors were inoculated on SC agar medium containing 80 mg/L 5-bromo-4-chloro-3-indolyl β-D-galactopyranoside, 2% (w/v) galactose, and 1% (w/v) raffinose and incubated at 30°C overnight.

#### Immunoblotting

Whole nematode lysates were prepared as previously described.^8^^,^^23^ Nematodes were grown to the L4 stage at either 20°C or 25°C on NGM plates seeded with OP50 and then incubated at 20°C for 24–48 h. Adult hermaphrodites were washed with M9 buffer three times and boiled in sodium dodecyl sulfate (SDS) sample buffer (62.5 mM Tris-HCl, pH 6.8, 2% [w/v] SDS, 10% [v/v] glycerol, 1% [v/v] 2-mercaptoethanol, and 0.01% [w/v] bromophenol blue) at 100°C for 10 min. The samples were subjected to SDS-PAGE and then transferred to nitrocellulose membranes. The membrane was blocked with 5% (w/v) skim milk in TBS-T buffer (5 mM Tris-HCl pH 8.0, 150 mM NaCl, and 0.05% [v/v] Tween 20) for 1 h and then incubated with primary antibody in TBS-T containing 5% (w/v) skim milk at 4°C overnight. After washing with TBS-T buffer three times, the membrane was incubated with secondary antibody in TBS-T containing 5% (w/v) skim milk at 25°C for 1 h. After washing with TBS-T buffer three times, SuperSignal West Pico PLUS Chemiluminescent Substrate (Thermo Fisher Scientific) was incubated on the membranes for 5 min, and the signal was detected with FUSION Solo 7 S (M&S Instruments). For stripping, the membrane was washed with TBS-T buffer and then incubated with Restore PLUS Western Blot Stripping Buffer (Thermo Fisher Scientific) for 15 min. The membrane was washed with TBS-T buffer and a second immunoprobing experiment was performed as described above.

The primary antibodies used in immunoblotting were goat polyclonal anti-GFP antibody (1:1000 dilution; Cat# 70R-GG001, RRID: AB_1286216, Fitzgerald Industries International) and mouse monoclonal anti-beta-actin antibody (1:2000–10,000 dilution; Cat# sc-47778, RRID: AB_626632, Santa Cruz Biotechnology). The secondary antibodies used in immunoblotting were peroxidase-conjugated donkey anti-goat immunoglobulin G antibody (1:5000 dilution; Cat# AP180P, RRID: RRID: AB_92573, Merck) and peroxidase-conjugated goat anti-mouse immunoglobulin G antibody (1:5000 dilution; Cat# 115-035-003, RRID: AB_10015289, Jackson ImmunoResearch).

#### Alignment of amino acid sequences

Amino acid sequences in *C*. *elegans* and other species were obtained from WormBase (https://wormbase.org/) and UniProt (https://www.uniprot.org/), respectively. Alignment of amino acid sequences was performed using ClustalX 2.1 (ref.^47^).

#### AlphaFold prediction

AlphaFold-predicted structures were obtained using AlphaFold Server (https://alphafoldserver.com/).^48^ Their visualization was performed using ChimeraX (version 1.10.1).^49^^,^^50^^,^^51^

#### Phylogenetic analysis

Phylogenetic analysis was performed, as described previously.^66^ Curation of alignment was performed using Gblocks (version 0.91.1; https://ngphylogeny.fr/tools/tool/276/form).^52^^,^^53^^,^^54^ Phylogenetic analysis was performed using PhyML 3.0 (http://www.atgc-montpellier.fr/phyml/)^55^ under the LG + G + F model, which was selected by Smart Model Selection in PhyML.^67^

### Quantification and statistical analysis

To quantify the area of persistent MOs and paternal mitochondria during embryogenesis, z-projection images were binarized and the signal area inside embryos was quantified using Fiji software.

To quantify the area of PGL granules in somatic cells, z-projection images were binarized, and the signal area in embryos excluding manually identified germ cell signals was quantified using Fiji software.

To quantify the intensity or morphology of GFP::BCAS-3 (Figures 4C, S4A, and S4B) and GFP::LGG-1 (Figures 6B and S6F) around paternal mitochondria in zygotes/embryos, single-plane images were analyzed to minimize the influence of adjacent signals derived from MOs. The intensity of GFP::BCAS-3, excluding Figures 4C and S4B, mCherry::BCAS-3, PHAF-1::GFP, and GFP::ATG-18 around paternal mitochondria in zygotes was quantified using Z-projection images. Regions of interest (ROIs) were manually drawn around paternal mitochondria, and the mean intensities of GFP and mCherry within each ROI were measured using Fiji software. When mitochondria formed inseparable clusters, they were analyzed collectively. Background intensity was subtracted using cytoplasmic regions of the same zygote, and the fluorescence from GFP- or mCherry-tagged BCAS-3, PHAF-1, or ATG-18 was normalized to mitochondrial fluorescence within the same ROI to account for z-axis-dependent variations in signal intensity.

Statistical analyses were performed using R software (version 4.5.1). Data normality was assessed with the Shapiro–Wilk test, and samples were considered nonparametric when the *p* value was less than 0.05. For PGL-1 granules, samples were treated as nonparametric without performing this test, because the total area of PGL granules in the wild type was zero for all samples. For parametric multiple comparisons, the homogeneity of variances was assessed using the Levene’s test. Samples were considered to have non-homogeneous variances when the *p* value was less than 0.05 Statistical differences between groups were determined by Welch’s one-way ANOVA followed by Games–Howell post hoc test. For nonparametric comparisons, the Mann–Whitney U test (with Holm adjustment for multiple comparisons) or the Kruskal–Wallis test followed by Dunn’s test with Holm adjustment was applied. Detailed information on statistical methods is indicated in each figure legend. Graphs were drawn using GraphPad Prism 11 software (GraphPad Software).
