# Supplementary material for: BCAS-3 is required for the progression of autophagosome formation to degrade paternal mitochondria in Caenorhabditis elegans
Source: iScience. 2026 Jun 11;29(7):116345. doi: 10.1016/j.isci.2026.116345 (PMC13276154; doi:10.1016/j.isci.2026.116345)
Supplement: Document S1. Figures S1–S6 and Table S1 [file mmc1.pdf]

iScience, Volume *29*

Supplemental information

BCAS-3 is required for the progression of autophagosome formation to degrade paternal mitochondria in *Caenorhabditis elegans*

Takuya Norizuki, Taeko Sasaki, Yuji Suehiro, Shohei Mitani, Waka Kojima, Koji Yamano, Noriyuki Matsuda, and Miyuki Sato

A

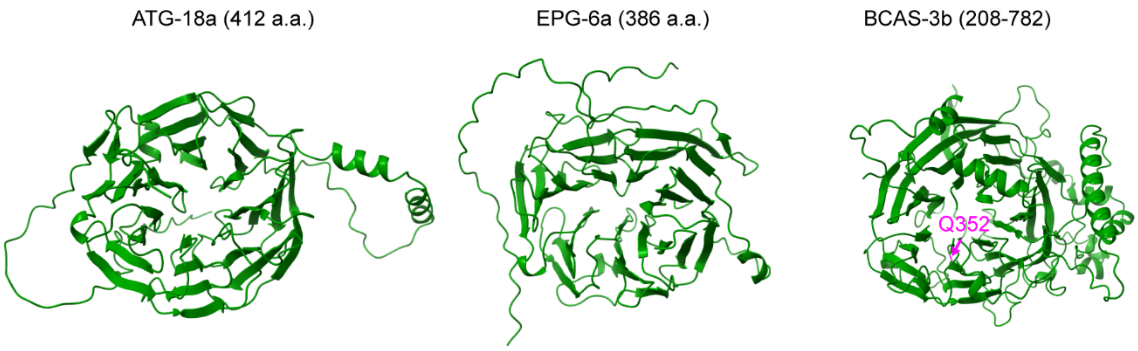
ATG-18a (412 a.a.)
EPG-6a (386 a.a.)
BCAS-3b (208-782)
Q352

B

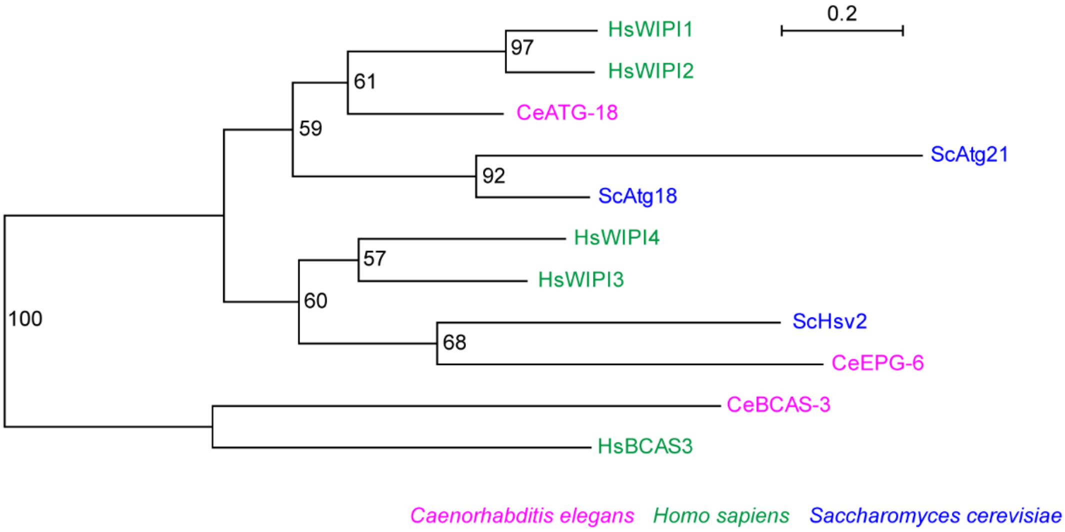
0.2
HsWIPI1
HsWIPI2
CeATG-18
ScAtg21
ScAtg18
HsWIPI4
HsWIPI3
ScHsv2
CeEPG-6
CeBCAS-3
HsBCAS3
97
61
59
92
57
60
68
100
Caenorhabditis elegans
Homo sapiens
Saccharomyces cerevisiae

Figure S1. Relationship between BCAS3 and PROPPIN proteins, related to Figure 1.

(**A**) AlphaFold-predicted structures of *C. elegans* ATG-18 (isoform a), EPG-6 (isoform a), and BCAS-3 (isoform b). For BCAS-3b (full length: 1031 a.a.), residues 208–782 corresponding to blades 1–7 of *Homo sapiens* BCAS3 (ref.[S1]) are shown. The Q352 residue in BCAS-3b, which is mutated in the *dk1* allele, is colored magenta. The structure of BCAS-3 (isoform b) is shown, since this isoform was used in yeast two-hybrid (**Figure 1F**).

(**B**) Maximum-likelihood phylogenetic tree of PROPPINs and BCAS3. Branch lengths indicate the estimated number of substitutions per site. Bootstrap values were calculated by resampling 1000 sets.

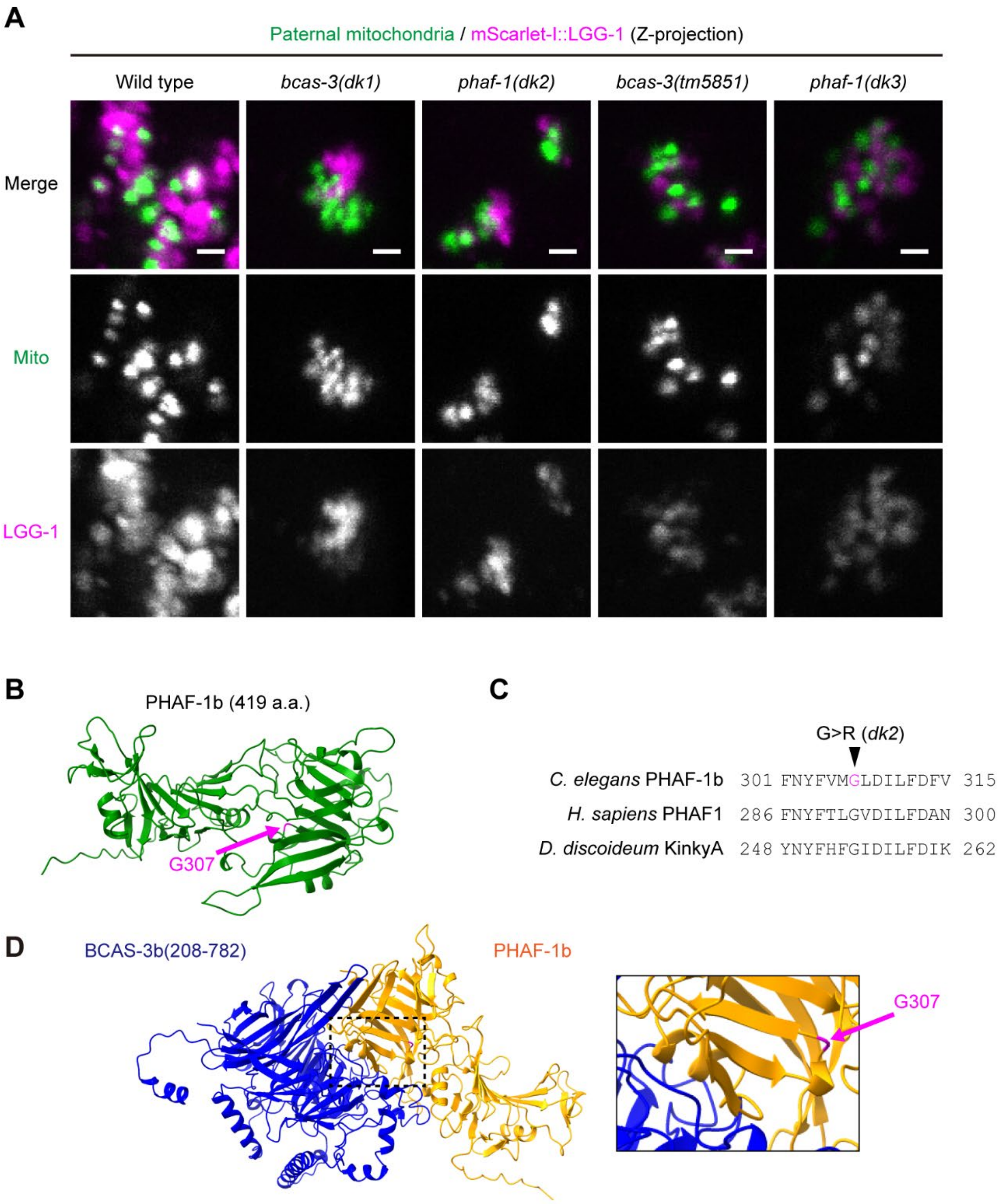
A
Paternal mitochondria / mScarlet-I::LGG-1 (Z-projection)
Wild type
bcas-3(dk1)
phaf-1(dk2)
bcas-3(tm5851)
phaf-1(dk3)
Merge
Mito
LGG-1
B
PHAF-1b (419 a.a.)
G307
C
G>R (dk2)
C. elegans PHAF-1b 301 FNYFVMGLDILFDFV 315
H. sapiens PHAF1 286 FNYFTLGVDILFDAN 300
D. discoideum KinkyA 248 YNYFHFGIDILFDIK 262
D
BCAS-3b(208-782)
PHAF-1b
G307

Figure S2. *bcas-3* and *phaf-1* mutants, related to Figure 1

(**A**) Z-projection images of the boxed regions in **Figure 1C**. Scale bars: 1 μm.

(**B**) AlphaFold-predicted structures of PHAF-1b. The position of G307, the residue mutated in the *dk2* allele, is colored in magenta. The structure of PHAF-1 (isoform b) is shown, since this isoform was used in the yeast two-hybrid assay (**Figure 1F**).

(**C**) Alignment of PHAF1 homologs in human, *C. elegans*, and *Dictyostelium discoideum*. The residue mutated in the *dk2* allele is colored magenta.

(**D**) AlphaFold-predicted structures of the BCAS-3 (blue) and PHAF-1 (orange) complex. The boxed region in the left panel is shown at higher magnification in the right panel. The position of G307, the residue mutated in the *dk2* allele, is colored magenta. The regions other than residues 208–782 in BCAS-3b are not shown because they are predicted to be unstructured.

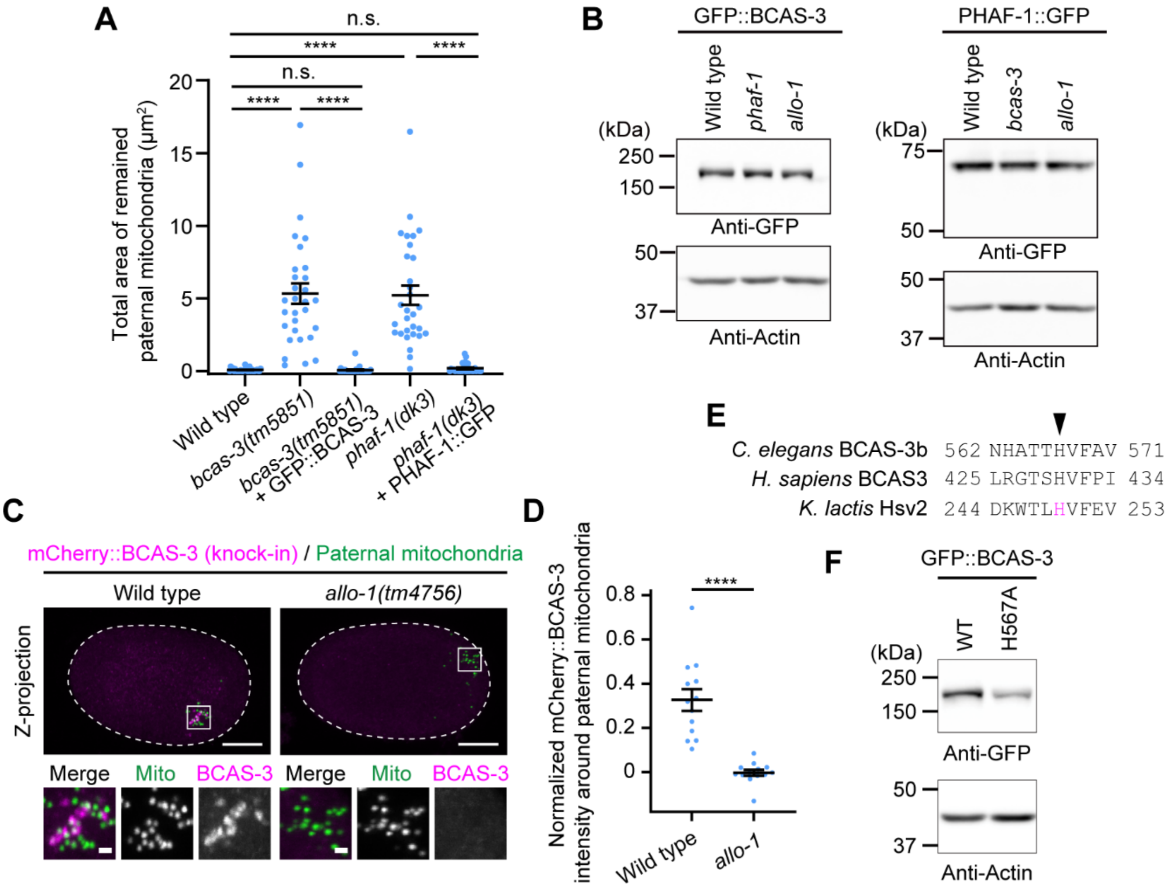
A
n.s.
****
****
n.s.
****
****
Total area of remained paternal mitochondria (μm²)
Wild type
bcas-3(tm5851)
bcas-3(tm5851) + GFP::BCAS-3
phaf-1(dk3)
phaf-1(dk3) + PHAF-1::GFP
B
GFP::BCAS-3
Wild type
phaf-1
allo-1
(kDa)
Anti-GFP
Anti-Actin
PHAF-1::GFP
bcas-3
C
mCherry::BCAS-3 (knock-in) / Paternal mitochondria
Wild type
allo-1(tm4756)
Z-projection
Merge
Mito
BCAS-3
D
Normalized mCherry::BCAS-3 intensity around paternal mitochondria
E
C. elegans BCAS-3b 562 NHATTHVFAV 571
H. sapiens BCAS3 425 LRGTSHVFPI 434
K. lactis Hsv2 244 DKWTLHVFEV 253
F
GFP::BCAS-3
WT
H567A

Figure S3. Localization of BCAS-3 and PHAF-1, related to Figure 3.

(**A**) Rescue experiment of *bcas-3*- or *phaf-1*-deficient zygotes by expression of GFP::BCAS-3 or PHAF-1::GFP. Persistent paternal mitochondria (HSP-6::mCherry) in the 32–64-cell stage embryos were quantified as the area showing a fluorescent signal per embryo. n= 30 embryos for each strain.

(**B**) Expression level of GFP::BCAS-3 and PHAF-1::GFP in the strains used in **Figures 3A–D**. Three independent experiments were performed, and representative images are shown.

(**C, D**) Recruitment of endogenous BCAS-3 to paternal mitochondria. The DNA fragment encoding mCherry was inserted before the start codon of *bcas-3* using the CRISPR-Cas9 system. The signal from mCherry::BCAS-3 was detected using an anti-mCherry antibody. The mCherry::BCAS-3 intensity around paternal mitochondria was normalized to the HSP-6::GFP (paternal mitochondria) intensity in each paternal mitochondrion, and the mean value in each zygote is shown. n = 13 zygotes from late meiosis I to pronuclear expansion. Dashed lines represent the outlines of the zygotes. Scale bars: 10 μm (upper panels) and 1 μm (lower panels).

(**E**) Alignment of BCAS3/BCAS-3 in *Homo sapiens* and *C. elegans*, and *Kluyveromyces lactis* PROPPIN, Hsv2. The residue important for PI3P binding in *K. lactis* Hsv2 (ref.[S2]) is colored magenta.

(**F**) Expression levels of GFP::BCAS-3^{WT} and GFP::BCAS-3^{H567A} in the strains used in **Figures 3E and F**. Three independent experiments were performed, and representative images are shown.

Error bars represent the mean ± SEM. Statistical differences were determined using the Mann–Whitney U test followed by Holm correction (**A**) or the Mann–Whitney U test (**D**). n.s.: $p > 0.05$; ****$p < 0.0001$.

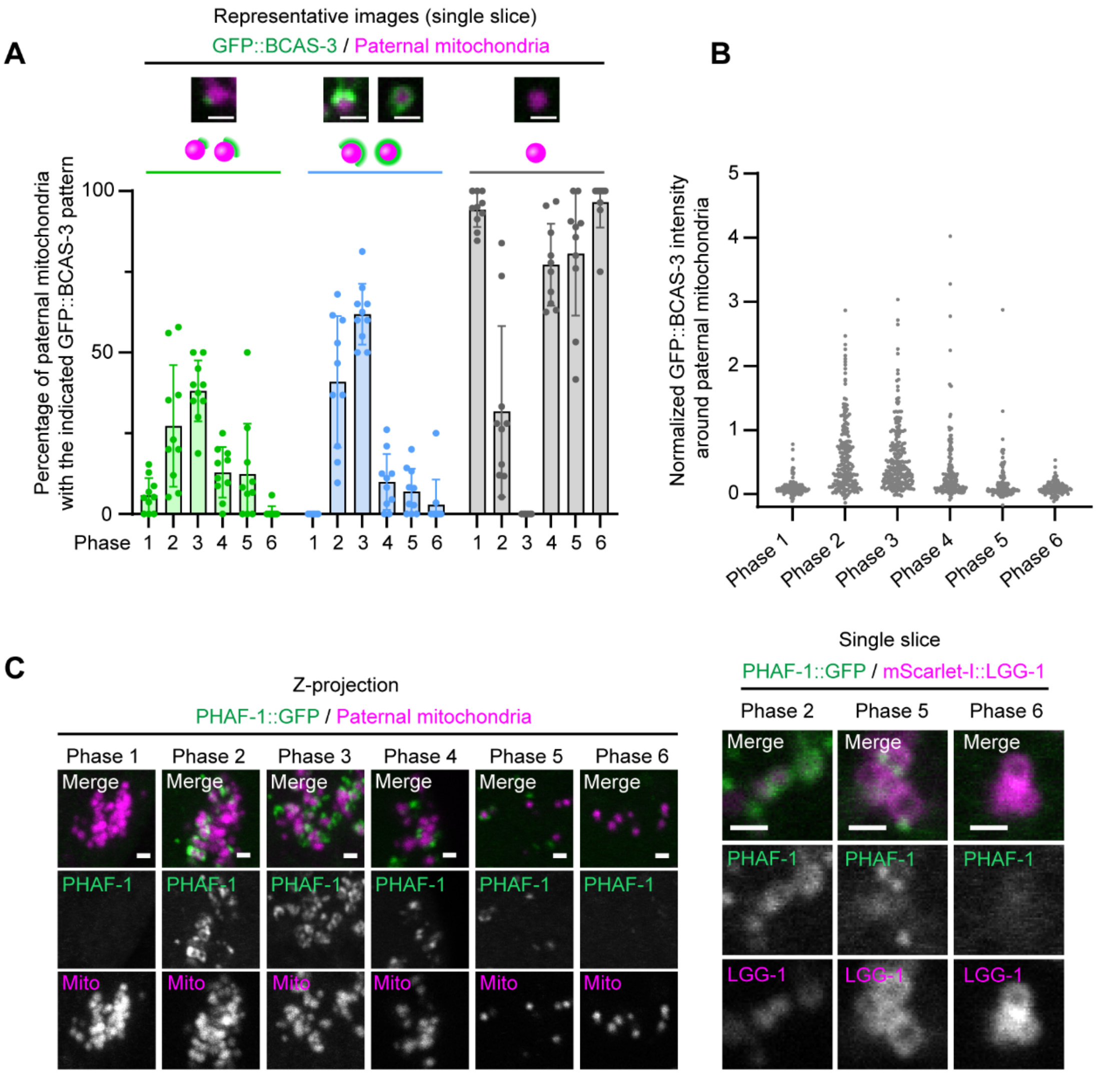
A
Representative images (single slice)
GFP::BCAS-3 / Paternal mitochondria
Percentage of paternal mitochondria with the indicated GFP::BCAS-3 pattern
100
50
0
Phase 1 2 3 4 5 6 1 2 3 4 5 6 1 2 3 4 5 6
B
Normalized GFP::BCAS-3 intensity around paternal mitochondria
5
4
3
2
1
0
Phase 1
Phase 2
Phase 3
Phase 4
Phase 5
Phase 6
C
Z-projection
PHAF-1::GFP / Paternal mitochondria
Phase 1
Phase 2
Phase 3
Phase 4
Phase 5
Phase 6
Merge
PHAF-1
Mito
Single slice
PHAF-1::GFP / mScarlet-I::LGG-1
Phase 2
Phase 5
Phase 6
Merge
PHAF-1
LGG-1

Figure S4. Temporal changes in PHAF-1::GFP signal around paternal mitochondria, related to Figure 4.

(**A**) Percentage of paternal mitochondria with the indicated GFP::BCAS-3 morphological patterns per zygote/embryo at each phase. Zygotes/embryos expressing GFP::BCAS-3 and HSP-6::mCherry (paternal mitochondria) were analyzed. Signals were classified as green when GFP surrounded less than half of the mitochondrion (puncta), blue when GFP surrounded half or more of the mitochondrion (cup- or ring-shaped structures), and gray when GFP was absent. Representative images of each morphological pattern are shown above the graph. Morphology was scored for each individual mitochondrion using the same dataset as in **Figure 4C** (10 zygotes/embryos per phase), and 127–238 structures per phase were analyzed. Error bars represent the mean ± SEM.

(**B**) GFP::BCAS-3 fluorescence intensities normalized to HSP-6::mCherry intensity around paternal mitochondria at each phase. Each data point represents a measurement from an individual mitochondrion, or when mitochondria could not be resolved, a mitochondrial cluster. These data were obtained from the same dataset used in **Figures 4C**; the corresponding mean fluorescence intensity per zygote/embryo is shown in **Figure 4C**.

(**C**) Representative images of PHAF-1::GFP and HSP-6::mCherry (paternal mitochondria) or mScarlet-I::LGG-1 in zygotes at each phase defined in **Figure 4A**. Scale bars: 1 μm.

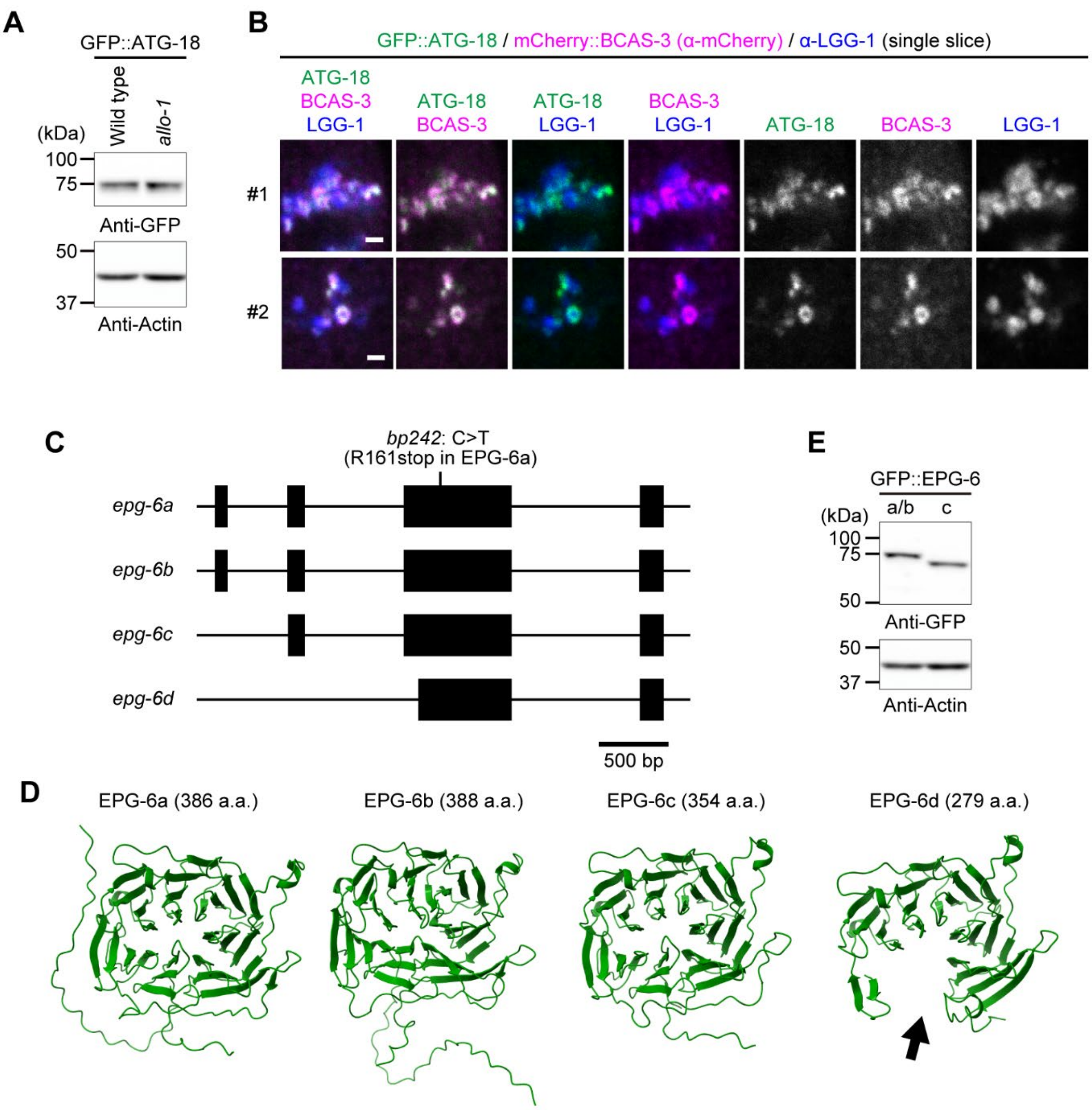
A
GFP::ATG-18
(kDa)
Wild type
allo-1
100
75
Anti-GFP
50
37
Anti-Actin
B
GFP::ATG-18 / mCherry::BCAS-3 (α-mCherry) / α-LGG-1 (single slice)
ATG-18
BCAS-3
LGG-1
#1
#2
C
bp242: C>T
(R161stop in EPG-6a)
epg-6a
epg-6b
epg-6c
epg-6d
500 bp
E
GFP::EPG-6
a/b
c
(kDa)
100
75
50
Anti-GFP
50
37
Anti-Actin
D
EPG-6a (386 a.a.)
EPG-6b (388 a.a.)
EPG-6c (354 a.a.)
EPG-6d (279 a.a.)

Figure S5. Localization of ATG-18 and EPG-6 during allophagy, related to Figure 5.

(**A**) Expression levels of GFP::ATG-18 in the strains used in **Figures 5A and B**. Three independent experiments were performed, and representative images are shown.

(**B**) Localization of ATG-18, BCAS-3, and LGG-1 during allophagy. The signals from GFP::ATG-18, endogenously mCherry-tagged BCAS-3 (α-mCherry), and endogenous LGG-1 (α-LGG-1) are shown in green, magenta, and blue, respectively. Eighteen zygotes from late meiosis I to pronuclear expansion were observed, and two representative images are shown. Scale bars: 1 μm.

(**C, D**) Four splice isoforms of the *epg-6* gene. Exons (**C**) and AlphaFold-predicted structures (**D**) of each isoform are shown. An arrow indicates incomplete seven-bladed β-propeller structures in EPG-6d.

(**E**) Expression levels of GFP::EPG-6 in the strains used in **Figure 5E**. Three independent experiments were performed, and representative images are shown.

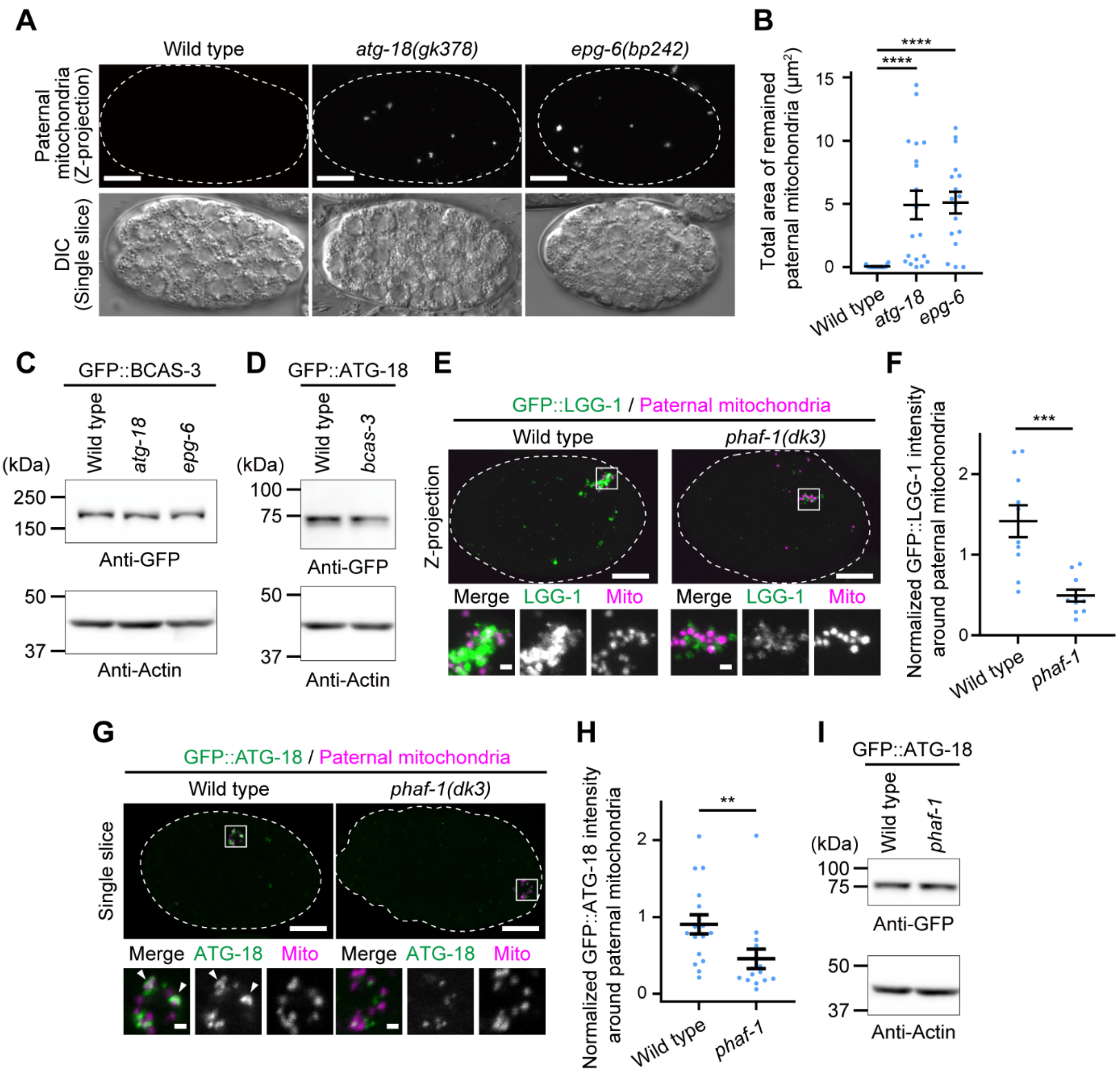
A
Wild type
atg-18(gk378)
epg-6(bp242)
Paternal mitochondria (Z-projection)
DIC (Single slice)
B
Total area of remained paternal mitochondria (μm²)
****
****
15
10
5
0
Wild type
atg-18
epg-6
C
GFP::BCAS-3
Wild type
atg-18
epg-6
(kDa)
250
150
Anti-GFP
50
37
Anti-Actin
D
GFP::ATG-18
Wild type
bcas-3
(kDa)
100
75
Anti-GFP
50
37
Anti-Actin
E
GFP::LGG-1 / Paternal mitochondria
Wild type
phaf-1(dk3)
Z-projection
Merge
LGG-1
Mito
Merge
LGG-1
Mito
F
Normalized GFP::LGG-1 intensity around paternal mitochondria
***
2
1
0
Wild type
phaf-1
G
GFP::ATG-18 / Paternal mitochondria
Wild type
phaf-1(dk3)
Single slice
Merge
ATG-18
Mito
Merge
ATG-18
Mito
H
Normalized GFP::ATG-18 intensity around paternal mitochondria
**
2
1
0
Wild type
phaf-1
I
GFP::ATG-18
Wild type
phaf-1
(kDa)
100
75
Anti-GFP
50
37
Anti-Actin

Figure S6. Functional relationship between BCAS-3 and WIPIs, related to Figure 6.

(**A, B**) Defects in degradation of paternal mitochondria in the *atg-18* and *epg-6* mutant. The total area of persistent paternal mitochondria was quantified. n = 18 (wild type), 19 (*atg-18*) and 17 (*epg-6*) embryos at 64-100 cell stages.

(**C, D**) Expression level of GFP::BCAS-3 (**C**) and GFP::ATG-18 (**D**) in the strains used in **Figures 6C–F**. Three independent experiments were performed, and representative images are shown.

(**E, F**) Impaired autophagosome formation in the *phaf-1* mutant. n = 10 zygotes per strain during pronuclear migration.

(**G, H**) Reduced accumulation of ATG-18 around paternal mitochondria in the *phaf-1* mutant. Arrowheads represent the elongated or cup-shaped structures. n = 17 (wild type) and 15 (*phaf-1*) zygotes from late meiosis I to pronuclear expansion.

(**I**) Expression level of GFP::ATG-18 in the strains used in **Figures S6G and H**. Three independent experiments were performed, and representative images are shown.

The GFP::LGG-1 (**F**) and GFP::ATG-18 (**H**) intensities around paternal mitochondria were normalized to the HSP-6::mCherry (paternal mitochondria) intensity in each paternal mitochondrion, and the mean value in each zygote is shown. Error bars represent the mean ± SEM. Statistical differences were determined using the Mann–Whitney U test followed by Holm correction (**B**) or the Mann–Whitney U test (**F** and **H**). $^{**}p < 0.01$; $^{***}p < 0.001$; $^{****}p < 0.0001$. Dashed lines represent the outlines of the zygotes. Scale bars: 10 μm (**A** and upper panels in **E and G**) and 1 μm (lower panels in **E and G**).

**Oligonucleotides**	**Sequence (5' -> 3')**
pID2.02_mScarlet-I_F	CAAATTTTCTTTTCCAGATGGTCTCCAAGGGAGAGGCC
pID2.02_mScarlet-I_R	TGGAAAATACGGCAAAATTACTTGTAGAGCTCGTCCATTCC
*lgg-1*_Infusion_F	gctgcaATGAAGTGGGCTTACAAGGA
*lgg-1*_Infusion_R	TGGAAAATACGGCAAAATTATTCCTTCTTTTCGACCTCTCC
pID2.02_Infusion_F	TAATTTTGCCGTATTTTCCATATT
pID2.02_Infusion_R	CATCTGGAAAAGAAAATTTGATTT
*bcas-3*_GWF	GGGGACAACTTTGTACAAAAAAGTTGtgccgccaaacgcgaactct
*bcas-3*_GWR	GGGGACAACTTTGTACAAGAAAGTTGtcaaatatcatccatgtcaa
*atg-18*_GWF	GGGGACAACTTTGTACAAAAAAGTTGtgtcggctacaacatcagaa
*atg-18*_GWR	GGGGACAACTTTGTACAAGAAAGTTGttagccgctggtgtggctca
*epg-6a/b*_GWF	GGGGACAACTTTGTACAAAAAAGTTGTGAGCAAGAAGGAGGAAACGATTTTC
*epg-6c*_GWF	GGGGACAACTTTGTACAAAAAAGTTGTGACAACGTTAAATCATGCATCAGTG
*epg-6*_GWR	GGGGACAACTTTGTACAAGAAAGTTGCTACTCGACTGGCGTACGGA
*bcas-3_H567A*_F	AACAACTGCCGTTTTCGCAGTTTG
*bcas-3_H567A*_R	AAAACGGCAGTTGTTGCGTGATTC
*phaf-1*_GWF	GGGGACAACTTTGTACAAAAAAGTTGtgaatggaaaacagcaagtc
*phaf-1*_GWR	GGGGACAACTTTGTACAAGAAAGTTGttatttttccttggctccatc
*phaf-1*_F1	aatgggattgGATATTCTGTTTGATTTTGTC
*phaf-1*_R1	acagaatatcCAATCCCATTACAAAGTAGT
pID2.02 Infusion-R1	CATCTGGAAAAGAAAATTTGATTT
pID2.02*epg7gfp*-F2	ATGAGTAAAGGAGAAGAACT
phaf-1-gfp-InfusionF	CAAATTTTCTTTTCCAGATGAATGGAAAACAGCAAGTC
phaf-1-gfp-InfusionR	AGTTCTTCTCCTTTACTCATTGCAGCTTTTTCCTTGGCTCCATCATA

*phaf-1*_G307R_F	TTGTAATGAGATTGGATATTCTGTTTGATTTTG
*phaf-1*_G307R_R	CCAATCTCATTACAAAGTAGTTGAAGAAG
*phaf-1*_ sgRNAoligoF1	ttgGAACTTTTTAGACCAACATC
*phaf-1*_ sgRNAoligoR1	aacGATGTTGGTCTAAAAAGTTC
*phaf-1*_ sgRNAoligoF2	ttggcagACGGAAGAATTGTCGA
*phaf-1*_ sgRNAoligoR2	aacTCGACAATTCTTCCGTctgc
*dpy-10*_ sgRNAoligo_F_SapTrap	ttgGCTACCATAGGCACCACGAG
*dpy-10*_ sgRNAoligo_R_SapTrap	aacCTCGTGGTGCCTATGGTAGC
*phaf-1*_ template_deletion	aatattttccaaagAAATTCAAGGTTGTACCAGATCGATGGAGAGTATGATGTATCCACGTACACACGAC
*dpy-10*_ cn64_repair template_70mer	CGGCAAGATGAGAATGACTGGAAACCGTACCGCATGCGGTGCCTATGGTAGCGGAGCTTCACATGGCTTC

Table S1. Oligonucleotides used in this study, related to STAR Methods

Supplemental reference

S1. Kojima, W., Yamano, K., Kosako, H., Imai, K., Kikuchi, R., Tanaka, K., and Matsuda, N. (2021). Mammalian BCAS3 and C16orf70 associate with the phagophore assembly site in response to selective and non-selective autophagy. Autophagy *17*, 2011-2036. 10.1080/15548627.2021.1874133.

S2. Baskaran, S., Ragusa, M.J., Boura, E., and Hurley, J.H. (2012). Two-site recognition of phosphatidylinositol 3-phosphate by PROPPINs in autophagy. Mol. Cell *47*, 339-348. 10.1016/j.molcel.2012.05.027.
